# Controlling LEF growth in some group extensions

**DOI:** 10.1007/s10801-026-01502-1

**Published:** 2026-02-26

**Authors:** Henry Bradford

**Affiliations:** Christ’s College, University of Cambridge, St Andrew’s Street, Cambridge, CB2 3BU UK

**Keywords:** LEF, Finitely presented groups, Schreier graphs

## Abstract

For a finitely generated LEF group $\Gamma$, we study the orders of finite groups admitting local embeddings of balls in a word metric on $\Gamma$, as measured by the *LEF growth function*. We prove that any sufficiently smooth increasing function between *n*! and $\exp (\exp (n))$ is close to the LEF growth function of some finitely generated group. This is achieved by estimating the LEF growth of some semidirect products of the form ${{\,\textrm{FSym}\,}}(\Omega ) \rtimes \Gamma$, where $\Omega \curvearrowleft \Gamma$ is an appropriate transitive action and ${{\,\textrm{FSym}\,}}(\Omega )$ is the group of finitely supported permutations of $\Omega$. A key tool in the proof is to identify sequences of finitely presented subgroups with short “relative” presentations. In a similar vein, we also obtain estimates on the LEF growth of some groups of the form $E_{\Omega } (R) \rtimes \Gamma$, for *R* an appropriate unital ring and $E_{\Omega } (R)$ the subgroup of ${{\,\textrm{Aut}\,}}_R (R[\Omega ])$ generated by all transvections with respect to basis $\Omega$.

## Introduction

A group $\Gamma$ is *LEF* if every finite region of its multiplication table appears in the multiplication table of some finite group *Q*. The class of LEF groups properly contains the class of residually finite groups. If $\Gamma$ is finitely generated by a subset *S*, then the balls $B_S(n)$ in the associated word metric on $\Gamma$ form a prototypical sequence of finite subsets. The *LEF growth function* $\mathcal {L}_{\Gamma } ^S$ of $\Gamma$ is defined such that $\mathcal {L}_{\Gamma } ^S (n) = |Q |$, where *Q* is of minimal order among finite groups in which the (partial) multiplication on $B_S (n)$ may be modeled. LEF growth was introduced in [1] and independently in [6] (under a different name). In this paper, we continue our study of the LEF growth function initiated in [7] and continued in [8].

### Statement of results

It is natural to ask which increasing functions arise as the LEF growth function of some group. For instance, in [7] were exhibited groups with LEF growth on the order of $n^d$, $\exp (n^d)$ (for every positive integer *d*) and $\exp (\exp (n))$. In [8], it was further shown that there are uncountably many inequivalent types of LEF growth. We improve on these results, using completely different methods. Indeed we show that almost any sufficiently smoothly increasing function between exponential and doubly exponential is close to the LEF growth function of some group. Since the dependence of the function $\mathcal {L}_{\Gamma } ^S$ on the choice of finite generating set *S* is slight, we suppress *S* from our notation for the rest of Introduction.

#### Theorem 1.1

Let $f: \mathbb {N} \rightarrow \mathbb {N}$ be a permissible function. Then there exists a finitely generated LEF group $\Delta (f)$ and constants $C,c>0$ such that for all *n*, $f(cn)! \le \mathcal {L}_{\Delta (f)} (n) \le f(Cn)!$.

Roughly, we call a function “permissable” if it is equivalent to the growth function of a connected regular graph (see Sect. 5). This is flexible enough to yield the following examples, among many others.

#### Corollary 1.2

For every $\alpha > 1$ and $\beta \in (0,1)$, there exist $C,c > 0$ and finitely generated LEF groups $\Gamma _1 (\alpha )$; $\Gamma _2 (\alpha )$ and $\Gamma _3 (\beta )$ such that for all *n*,

(i) $$\exp (c n^{\alpha }) \le \mathcal {L}_{\Gamma _1 (\alpha )}(n) \le \exp (C n^{\alpha })$$;
(ii) $$\exp \big (c \exp (\log (cn)^{\alpha })\big ) \le \mathcal {L}_{\Gamma _2 (\alpha )}(n) \le \exp \big (C \exp (\log (Cn)^{\alpha })\big )$$;
(iii) $$\exp \big (c \exp (cn^{\beta })\big ) \le \mathcal {L}_{\Gamma _3 (\beta )}(n) \le \exp \big (C \exp (Cn^{\beta })\big )$$.

Let us begin to describe how the groups in Theorem 1.1 are constructed. One attractive feature of the class of LEF groups, which is not shared by the class of residually finite groups, is that LEF is preserved under various natural classes of group extensions, while residual finiteness is not. To wit, for any groups $\Gamma$ and $\Delta$, a $\Gamma$-set $\Omega$, and any unital ring *R*, $\Gamma$ acts naturally by automorphisms on (i) the direct sum $\oplus _{\Omega } \Delta$; (ii) the group ${{\,\textrm{FSym}\,}}(\Omega )$ of finitely supported permutations of the set $\Omega$; and (iii) the subgroup $E_{\Omega } (R)$ of ${{\,\textrm{Aut}\,}}_R (R[\Omega ])$ generated by all transvections with respect to $\Omega$ (where $R[\Omega ]$ is the free *R*-module on basis $\Omega$). We therefore have corresponding semidirect products: (i) the *wreath product* $\Delta \wr _{\Omega } \Gamma = (\oplus _{\Omega } \Delta ) \rtimes \Gamma$; (ii) the *symmetric enrichment* $\mathcal {S}(\Gamma ,\Omega ) = {{\,\textrm{FSym}\,}}(\Omega ) \rtimes \Gamma$; and (iii) the *elementary enrichment* $\mathcal {E}(\Gamma ,\Omega ,R) = E_{\Omega } (R) \rtimes \Gamma$. If $\Gamma$ and $\Delta$ are finitely generated LEF groups and the action of $\Gamma$ on $\Omega$ is a transitive *LEF action* (see Definition 2.7), then the groups $\Delta \wr _{\Omega } \Gamma$, $\mathcal {S}(\Gamma ,\Omega )$, $\mathcal {E}(\Gamma ,\Omega ,\mathbb {Z})$ and $\mathcal {E}(\Gamma ,\Omega ,\mathbb {F}_p)$ are finitely generated LEF groups. In this paper, we study the LEF growth of symmetric and elementary enrichments (wreath products having already been studied in [7]). To give a sample of our conclusions, in the special case for which $\Omega = \Gamma$ with the regular $\Gamma$-action, we have the following bounds (see Theorems 3.3 and 4.5 for the full statements encompassing non-regular transitive LEF actions).

#### Theorem 1.3

Let $\Gamma$ be a finitely generated LEF group. There exist $C,c > 0$ such that for all *n*,

(i) $$\gamma _{\Gamma }(cn)! \le \mathcal {L}_{\mathcal {S}(\Gamma ,\Gamma )} (n) \le \mathcal {L}_{\Gamma } (Cn)!$$;
(ii) $$\exp \big (cn \gamma _{\Gamma } (cn^{1/2})^2 \big ) \le \mathcal {L}_{\mathcal {E}(\Gamma ,\Gamma ,\mathbb {Z})} (n) \le \exp \big ( Cn \mathcal {L}_{\Gamma }(Cn)^2 \big )$$;
(iii) $$\exp \big (c\gamma _{\Gamma } (cn^{1/2})^2 \big ) \le \mathcal {L}_{\mathcal {E}(\Gamma ,\Gamma ,\mathbb {F}_p)} (n) \le \exp \big (C \mathcal {L}_{\Gamma }(Cn)^2 \big )$$.

Here $\gamma _{\Gamma }$ is the word growth function of $\Gamma$. In particular, if $\mathcal {L}_{\Gamma }$ is comparable to $\gamma _{\Gamma }$, then $\mathcal {L}_{\mathcal {S}(\Gamma ,\Gamma )}$ is known, up to an appropriate notion of equivalence of functions. Examples of groups $\Gamma$ for which this is the case include all finitely generated abelian groups and all finitely generated linear groups which are not virtually nilpotent.

The groups $\Delta (f)$ arising in Theorem 1.1 shall have the form $\mathcal {S}(\Gamma ,\Omega (f))$, for $\Gamma$ a non-abelian free group and $\Omega (f)$ an infinite set which we equip with a transitive LEF $\Gamma$-action such that the growth function of the corresponding Schreier graph is precisely *f*.

### Background and methods

The LEF growth function $\mathcal {L}_{\Gamma }$ may be compared with the *full residual finiteness growth function* $\mathcal {R}_{\Gamma }$; see Sect. 2.2. We always have $\mathcal {L}_{\Gamma } \le \mathcal {R}_{\Gamma }$, and the two functions are equivalent when $\Gamma$ is finitely presented. The function $\mathcal {R}_{\Gamma }$ has been extensively studied in a series of papers starting with [4], and good estimates are known for many families of groups. On the other hand, it is not well understood which functions *f* which arise as $\mathcal {R}_{\Gamma }$ (up to equivalence) for some finitely generated group $\Gamma$: Polynomials of any (integral) degree and the exponential function can arise, as can certain very quickly growing functions (see [5]) but beyond this not much is known. By contrast, our results provide a wide spectrum of LEF growth functions of finitely generated groups, in the range between exponential and doubly exponential functions. The groups arising in Theorem 1.1 are not residually finite, and it would be of interesting to investigate constructions of groups exhibiting a broader range of full residual finiteness growth functions than have hitherto been observed.

Local embeddings of elementary and symmetric enrichments into finite groups have been exploited in the context of several problems in geometric group theory. Vershik and Gordon [16] considered the group $\mathcal {S}(\Gamma ,\Gamma )$ in the case $\Gamma = \mathbb {Z}$, and showed that it is LEF. Later Elek and Szabo [11] used symmetric enrichments to construct the first examples of sofic (indeed LEF) groups which are not residually amenable. Properties of the local embeddings of elementary enrichments were studied by Mimura and Sako in connection with constructions of exotic box spaces in coarse geometry [14], and were used in [13] to exhibit dense subgroups of a common profinite group with radically different coarse properties.

Upper bounds on the LEF growth of wreath products; symmetric enrichments and elementary enrichments follow from the same “natural” elementary constructions which witness that these extensions preserve LEF. That is to say, if $Q_{\Gamma }$ and $Q_{\Delta }$ are finite groups, admitting local embeddings from appropriate subsets of $\Gamma$ and $\Delta$, and $q \in \mathbb {N}$ is sufficiently large, then balls in $\Delta \wr \Gamma$, $\mathcal {S}(\Gamma )$, $\mathcal {E}(\Gamma ,\mathbb {Z})$, and $\mathcal {E}(\Gamma ,\mathbb {F}_p)$ admit local embeddings into $Q_{\Delta } \wr Q_{\Gamma }$, $\mathcal {S}(Q_{\Gamma })$, $\mathcal {E}(Q_{\Gamma },\mathbb {Z}/q\mathbb {Z})$, and $\mathcal {E}(Q_{\Gamma },\mathbb {F}_p)$, respectively (and similarly for the extensions corresponding to non-regular LEF actions).

More challenging are lower bounds on LEF growth. Our approach here exploits the equivalence of LEF and residual finiteness within the class of *finitely presented* groups. For LEF groups which are *not* finitely presented (as most of the examples we consider will not be), this equivalence can still be useful when applied on subgroups. In other words, by identifying a sequence $(H_n)$ of finitely presented subgroups of a group $\Gamma$, we extract useful lower bounds on $\mathcal {L}_{\Gamma }$ from lower bounds on the full residual finiteness growth functions $\mathcal {R}_{H_n}$ (at various radii; see Proposition 2.15 for the relevant technical statement). In symmetric and elementary enrichments, the groups $H_n$ will be of the form ${{\,\textrm{Sym}\,}}(k_n)$ and ${{\,\textrm{SL}\,}}_{k_n} (\mathbb {Z})$ or ${{\,\textrm{SL}\,}}_{k_n} (\mathbb {F}_p)$, respectively (for appropriate increasing sequences of positive integers $(k_n)$).

For the sake of comparison with Theorem 1.3, an upper bound on the LEF growth of a regular wreath product of two LEF groups was proved in [1], and in [7], a complementary lower bound was given in a special case.

#### Theorem 1.4

([7] Theorem 1.9) Let $\Gamma$ be a finitely generated LEF group, and let $\Delta$ be a finite non-trivial group with trivial center. Then,

$$\begin{aligned} \exp \big ( \gamma _{\Gamma } (n) \big ) \preceq \mathcal {L}_{\Delta \wr _{\Gamma } \Gamma }(n) \preceq \exp \big ( \mathcal {L}_{\Gamma }(n) \big )\text {.} \end{aligned}$$

The lower bound in Theorem 1.4 uses different methods from the present paper, being based on the fact that one can recognize locally whether a family of finite centerless subgroups generates their direct product. We do not treat the case of the wreath product $\Delta \wr _{\Omega } \Gamma$ in detail here, although the methods of [1, 7] would allow one to generalize the bounds of Theorem 1.4 to these groups for non-regular LEF actions, at least when $\Delta$ is finite centerless.

The paper is structured as follows: In Sect. 2, we detail basic properties of the LEF growth function; discuss the relationship between LEF growth and full residual finiteness growth; and connect this relationship to finite presentability. We also record some finite presentations of ${{\,\textrm{Sym}\,}}(n)$, ${{\,\textrm{SL}\,}}_n(\mathbb {Z})$, and ${{\,\textrm{SL}\,}}_n(p)$, to be used subsequently. In Sects. 3 and 4, we prove our bounds on the LEF growth of symmetric enrichments and elementary enrichments of LEF groups, respectively. In Sect. 5, we prove Theorem 1.1.

### Basic notation and terminology

An action of a group $\Gamma$ on a set $\Omega$ will be denoted $\Omega \curvearrowleft \Gamma$. Unless specified, all our actions will be on the right. For $S \subseteq \Gamma$, the associated *Schreier graph* ${{\,\textrm{Schr}\,}}(\Gamma ,\Omega ,S)$ is the directed graph with vertices $\Omega$ and edges $\Omega \times S$, with $\iota (\omega , s) = \omega$ and $\tau (\omega , s)=\omega s$. We regard the edge $(\omega , s)$ as being labeled by the element $s\in S$. For $n \in \mathbb {N}$ and $\omega \in \Omega$, $B_{S,\omega }(n) \subseteq \Omega$ denotes the closed ball of radius *n* around $\omega$ in the path metric on ${{\,\textrm{Schr}\,}}(\Gamma ,\Omega ,S)$. We shall also denote by $B_{S,\omega }(n)$ the induced subgraph of ${{\,\textrm{Schr}\,}}(\Gamma ,\Omega ,S)$ on this set. In the special case of $\Omega = \Gamma$ with the regular action, ${{\,\textrm{Schr}\,}}(\Gamma ,\Omega ,S)={{\,\textrm{Cay}\,}}(\Gamma ,S)$ is the *Cayley graph* and we write $B_S(n)$ for $B_{S,1}(n)$.

## Preliminaries

This section summarizes much of the basic material which was treated in greater detail in [7][Section 2]. There is also significant overlap with the relevant parts of [1].

### Definition and first properties

#### Definition 2.1

For $\Gamma ,\Delta$ groups and $F \subseteq \Gamma$, a function $\pi : F \rightarrow \Delta$ is a *partial homomorphism* if, for all $g,h \in F$, if $gh \in F$, then $\pi (gh)=\pi (g)\pi (h)$. An injective partial homomorphism will be called a *local embedding*. The group $\Gamma$ is *locally embeddable into finite groups (LEF)* if, for all finite $F \subseteq \Gamma$, there exist a finite group *Q* and a local embedding of *F* into *Q*.

#### Remark 2.2

The restriction of a local embedding $\pi : F \rightarrow \Delta$ to any subset $F^{\prime } \subseteq F$ is also a local embedding.

Subsequently we assume that $\Gamma$ is generated by the finite set *S*.

#### Definition 2.3

The *LEF growth* of $\Gamma$ (with respect to *S*) is:

$$\begin{aligned} \mathcal {L}_{\Gamma } ^S (n) = \min \lbrace |Q |: \exists \pi :B_S(n)\rightarrow Q\text { a local embedding} \rbrace \end{aligned}$$

(with $\mathcal {L}_{\Gamma } ^S (n) = \infty$ if the set on the right-hand side is empty).

#### Definition 2.4

For $f_1, f_2: (0,\infty ) \rightarrow (0,\infty )$, write $f_1 \preceq f_2$ if, for some constant $C>0$, $f_1(x) \le f_2 (Cx)$ for all $x \in (0,\infty )$. We write $f_1 \approx f_2$ and say $f_1$ *is equivalent to* $f_2$ if $f_1 \preceq f_2$ and $f_2 \preceq f_1$. By linear extension on each interval $[n,n+1]$, we may also compare functions defined on $\mathbb {N}$ under the relations $\preceq$ and $\approx$.

See Remark 2.6 of [7] for comparison of this notion of equivalence of functions to others used in the literature.

#### Lemma 2.5

Let $\Delta \le \Gamma$ be finitely generated by $S^{\prime }$. Let $M \in \mathbb {N}$ be such that $S^{\prime } \subseteq B_S (M)$. Then for all *n*, $\mathcal {L}_{\Delta } ^{S^{\prime }} (n) \le \mathcal {L}_{\Gamma } ^S (Mn)$. In particular, $\mathcal {L}_{\Delta } ^{S^{\prime }} \preceq \mathcal {L}_{\Gamma } ^S$.

Consequently, the LEF growth only depends on a choice of generating set up to equivalence.

#### Corollary 2.6

Let $S,S^{\prime } \subseteq \Gamma$ be finite generating sets. Let $L,M \in \mathbb {N}$ be such that $S^{\prime } \subseteq B_S (L)$ and $S \subseteq B_{S^{\prime }} (M)$. Then,

$$\begin{aligned} \mathcal {L}_{\Gamma } ^S (n) \le \mathcal {L}_{\Gamma } ^{S^{\prime }} (Mn) \le \mathcal {L}_{\Gamma } ^S (LMn). \end{aligned}$$

In particular $$\mathcal {L}_{\Gamma } ^S \approx \mathcal {L}_{\Gamma } ^{S^{\prime }}$$.

#### Definition 2.7

An action $\Omega \curvearrowleft \Gamma$ of a group $\Gamma$ on a set $\Omega$ is a *LEF action* if, for all finite subsets $F \subseteq \Gamma$ and $\Sigma \subseteq \Omega$, there exist an action $X \curvearrowleft \Delta$ of a finite group $\Delta$ on a finite set *X*; a partial homomorphism $\pi : F \rightarrow \Delta$ and an injective map $\theta : \Sigma \rightarrow X$ satisfying:

1 $$\begin{aligned} {\text {for all }} g \in F {\text { and }} \omega \in \Sigma , {\text { if }} \omega g\in \Sigma {\text { then }} \theta (\omega g)=\theta (\omega )\pi (g). \end{aligned}$$

We call the pair $(\pi ,\theta )$ a *partial morphism* of actions. If moreover $\pi$ is injective, we call $(\pi ,\theta )$ a *local embedding* of $(F,\Sigma )$ into $(\Delta ,X)$.

#### Remark 2.8

In the definition of a LEF action $\Omega \curvearrowleft \Gamma$, we do not require the partial homomorphism $\pi : F \rightarrow \Delta$ to be injective. However, given $\pi : F \rightarrow \Delta$ and $\theta : \Sigma \rightarrow X$ a partial morphism of actions as in Definition 2.7, and a local embedding $\phi : F \rightarrow Q$, for *Q* some finite group, we may let $\Delta ' = \Delta \times Q$ act on *X* via $x(h,q)=xh$ (for $h \in \Delta$, $q \in Q$ and $x \in X$) and define $\pi ': F \rightarrow \Delta '$ by $\pi ' (g) = (\pi (g),\phi (g))$. Then $(\pi ',\theta )$ is a local embedding of $(F,\Sigma )$ into $(\Delta ',X)$.

Thus, under the assumption that $\Gamma$ is a LEF group, an action $\Omega \curvearrowleft \Gamma$ of $\Gamma$ on a set $\Omega$ is LEF iff for all finite subsets $F \subseteq \Gamma$ and $\Sigma \subseteq \Omega$, there exist an action $X \curvearrowleft \Delta$ of a finite group $\Delta$ on a finite set *X* and a local embedding $(\pi ,\theta )$ of $(F,\Sigma )$ into $(\Delta ,X)$.

#### Example 2.9

If $F \subseteq \Gamma$ and $\pi : F \rightarrow \Delta$ is a local embedding, then $(\pi ,\pi )$ is a local embedding of (*F*, *F*) into $(\Delta ,\Delta )$ (with respect to the regular actions $\Gamma \curvearrowleft \Gamma$ and $\Delta \curvearrowleft \Delta$). As such, if $\Gamma$ is a LEF group, then the regular action $\Gamma \curvearrowleft \Gamma$ is a LEF action.

More generally, if $H \le \Gamma$ is closed in the profinite topology on $\Gamma$ and has trivial normal core, then for any finite $F \subseteq \Gamma$ and $\Sigma \subseteq H\backslash \Gamma$, there exists $H \le \overline{H} \le \Gamma$, with $|\Gamma :H|< \infty$, such that the natural maps $\Gamma \rightarrow {{\,\textrm{Sym}\,}}(\overline{H}\backslash \Gamma )$ and $H\backslash \Gamma \rightarrow \overline{H}\backslash \Gamma$ induce a local embedding of $(F,\Sigma )$ into $({{\,\textrm{Sym}\,}}(\overline{H}\backslash \Gamma ),\overline{H}\backslash \Gamma )$.

#### Example 2.10

Suppose $\Gamma$ is a split extension with kernel *K* and quotient *Q*. If $\Gamma$ is LEF then the action of *Q* on *N* by conjugation is LEF.

A further interesting construction of LEF actions will be given in Remark 3.12 (ii).

### LEF, residual finiteness and finite presentability

We recall that $\Gamma$ is *residually finite (RF)* if, for every finite subset $F \subset \Gamma$, there exists a finite group *Q* and a homomorphism $\pi : \Gamma \rightarrow Q$ such that the restriction $\pi |_F$ of $\pi$ to *F* is injective. If $\Gamma$ is generated by the finite set *S*, one may make the following definition (see [4]).

#### Definition 2.11

The *(full) RF growth* of $\Gamma$ is:

$$\begin{aligned} \mathcal {R}_{\Gamma } ^S (n) = \min \lbrace |F |: \exists \pi : \Gamma \rightarrow F {\text { s.t. }} \pi |_{B_S(n)} {\text { is injective}} \rbrace . \end{aligned}$$

The next observation is almost immediate from these definitions.

#### Lemma 2.12

If $\Gamma$ is residually finite, then it is LEF, and $\mathcal {L}_{\Gamma } ^S (n) \le \mathcal {R}_{\Gamma } ^S (n)$ for all *n*.

Among *finitely presented* groups we have a converse inequality (see [7] Proposition 2.18).

#### Proposition 2.13

If $\Gamma$ is finitely presented and LEF, then it is residually finite. Moreover, if $\Gamma \cong \langle S \mid R \rangle$ is a finite presentation with $R \subseteq F(S)$ consisting of reduced words of length at most $n_0$, then $\mathcal {R}_{\Gamma } ^S (n) = \mathcal {L}_{\Gamma } ^S (n)$ for all $n \ge n_0$.

#### Corollary 2.14

Suppose $\mathcal {R}_{\Gamma } ^S (n) \ne \mathcal {L}_{\Gamma } ^S (n)$ for infinitely many *n*. Then $\Gamma$ is not finitely presentable.

The following “relative” variant of Proposition 2.13 will be one of our main tools for proving non-trivial lower bounds on LEF growth in examples.

#### Proposition 2.15

Let $\Gamma$ be finitely generated by *S*. For $n \in \mathbb {N}$, let $H_n \le \Gamma$, and let $S^{\prime } _n \subseteq S^{\prime \prime } _n \subseteq H_n$ be finite subsets. Suppose that $H_n$ is finitely presented by $\langle S^{\prime } _n \mid R_n \rangle$, and let $f,g: \mathbb {N} \rightarrow \mathbb {N}$ be such that for all $n \in \mathbb {N}$:

$$\begin{aligned} S^{\prime \prime } _n&\subseteq B_S (f(n)); \\ R_n&\subseteq B_{S^{\prime } _n} (g(n)){\text { (in }}F(S^{\prime } _n)\text {).} \end{aligned}$$

Then $$\mathcal {L}_{\Gamma } ^S (f(n)g(n)) \ge \mathcal {R}_{H_n} ^{S^{\prime \prime } _n} (g(n))$$.

#### Proof

Viewing $S^{\prime } _n$ as a subset of *F*(*S*), we have $R_n \subseteq B_S (f(n)g(n))$. Let *Q* be a finite group and $\pi : B_S (f(n)g(n)) \hookrightarrow Q$ be a local embedding. Then since $e_{\Gamma } \in B_S (f(n)g(n))$, $\pi (R_n) = \lbrace e_Q \rbrace$, so $\pi \mid _{S^{\prime } _n}$ extends to a homomorphism $\tilde{\pi }: H_n \rightarrow Q$, which agrees with $\pi$ on $B_{S^{\prime \prime } _n} (g(n)) \subseteq B_S (f(n)g(n))$. In particular, the restriction of $\tilde{\pi }$ to $B_{S^{\prime \prime } _n} (g(n))$ is injective, so $|Q |\ge \mathcal {R}_{H_n} ^{S^{\prime \prime } _n} (g(n))$. $\square$

### LEF growth and growth of Schreier graphs

Let $\Gamma$ be finitely generated by *S*, and suppose $\Omega \curvearrowleft \Gamma$. For $\omega _0 \in \Omega$, the *growth function* $\gamma _{\Gamma ,\Omega } ^{S,\omega _0}: \mathbb {N}\rightarrow \mathbb {N}$ of the associated Schreier graph ${{\,\textrm{Schr}\,}}(\Gamma ,\Omega ,S)$ (with respect to *S* and $\omega _0$) is given by $\gamma _{\Gamma ,\Omega } ^{S,\omega _0} (n)= |B_{S,\omega _0}(n)|$. If $\Omega \curvearrowleft \Gamma$ is transitive, it is not difficult to see that the $\approx$-class of $\gamma _{\Gamma ,\Omega } ^{S,\omega _0}$ is independent of *S* and $\omega _0$; we may write $\gamma _{\Gamma ,\Omega }$ for this class. In the special case of the regular action $\Gamma \curvearrowleft \Gamma$, we recover the *word growth* function $\gamma _{\Gamma } ^S$ of $\Gamma$ (with respect to *S*); we write $\gamma _{\Gamma }$ for its $\approx$-class.

#### Remark 2.16

If there is a local embedding $B_S (n) \rightarrow Q$, then $|B_S (n) |\le |Q |$, so $\gamma _{\Gamma } ^S (n) \le \mathcal {L}_{\Gamma } ^S (n)$.

#### Definition 2.17

The group $\Gamma$ is *efficiently locally embeddable in finite groups* (ELEF) if $\gamma _{\Gamma } \approx \mathcal {L}_{\Gamma }$.

#### Example 2.18

(i) $\mathbb {Z}^k$ is ELEF; $\mathcal {L}_{\mathbb {Z}^k}(n) \approx n^k$ [7][Example 2.24];
(ii) A finitely generated virtually nilpotent group is ELEF iff it is virtually abelian [7][Proposition 2.26];
(iii) Let $\Gamma$ be a finitely generated group admitting a faithful linear representation over a field, and suppose $\Gamma$ is not virtually nilpotent. Then it is well known that $\gamma _{\Gamma }$ is exponential. Meanwhile, by [3][Theorem 1.1] $\mathcal {R}_{\Gamma }$ (called in that paper *residual girth*) is at most exponential. It follows that $\Gamma$ is ELEF, and $\mathcal {L}_{\Gamma } (n) \approx \exp (n)$ [7][Example 2.25];
(iv) Since every non-abelian finite rank free group *F* is linear over $\mathbb {Z}$ and is not virtually nilpotent, $\Gamma =F$ satisfies the conclusion of (iii).

### Group presentations

In order to extract good lower bounds on $\mathcal {L}_{\Gamma } ^S$ from Proposition 2.15 in Sects. 3 and 4, it will be important that the subgroups $H_n$ admit presentations with relator sets consisting of short words.

#### The symmetric group

The presentations of the finite symmetric groups, with a generating set consisting of transpositions, were studied in [15], where the following Theorem was proved.

##### Theorem 2.19

Let $T=(V,E)$ be a finite tree. For $e = (v,w) \in E$, let $s_{e} = (v \; w) \in {{\,\textrm{Sym}\,}}(V)$. Then $S_T = \lbrace s_{e}: e \in E \rbrace$ generates ${{\,\textrm{Sym}\,}}(V)$, with defining presentation $\big \langle S_T \mid R_T \big \rangle$, where $R_T = R_T ^1 \cup R_T ^2 \cup R_T ^3 \cup R_T ^4$ and

$$\begin{aligned} R_T ^1&= \lbrace s_e ^2 : e \in E \rbrace \\ R_T ^2&= \lbrace (s_{(v,w)} s_{(x,y)})^2 : (v,w),(x,y) \in E; v,w,x,y \in V \text { distinct} \rbrace \\ R_T ^3&= \lbrace (s_{(v,w)} s_{(v,x)})^3 : (v,w),(v,x) \in E; w \ne x \rbrace \\ R_T ^4&= \lbrace (s_{(v,w)} s_{(v,x)} s_{(v,w)} s_{(v,y)})^2 : (v,w),(v,x),(v,y) \in E; v,w,x,y \in V \text { distinct} \rbrace \text {.} \end{aligned}$$

Note that the special case of Theorem 2.19 for which *T* is a line segment is precisely the Coxeter presentation for ${{\,\textrm{Sym}\,}}(V)$.

##### Remark 2.20

It is clear that $\lbrace s_e: e \in E \rbrace$ generates ${{\,\textrm{Sym}\,}}(V)$. Let $v, w \in V$. Let $v = v_0, v_1, \ldots , v_l = w$ be a path in *T* from *v* to *w*. Then,

$$\begin{aligned} (v \; w) = s_{(v_1,v_2)} ^{s_{(v_0,v_1)}\cdots s_{(v_{l-1},v_l)}} \end{aligned}$$

so all transpositions are generated by $\lbrace s_e: e \in E \rbrace$, as required. Moreover, it is not difficult to see that the relations $R_T$ between the $S_T$ hold in ${{\,\textrm{Sym}\,}}(V)$. To prove Theorem 2.19, it therefore suffices to check that $R_T$ is a sufficient set of relations between the generators $S_T$.

#### The special linear group

Let *R* be a commutative ring with unity. For *V* a countable set, let $R^V$ be the free *R*-module (with *V* a free basis). Given $v,w\in V$ distinct elements and $r \in R$, define the *transvection* $E_{v,w} (r) \in {{\,\textrm{Aut}\,}}_R (R^V)$ by:

$$\begin{aligned} E_{v,w} (r): v\mapsto v+rw{\text { and }}E_{v,w} (r): x\mapsto x~({\text {for}}~ v \ne x \in V). \end{aligned}$$

For $v,w,x,y \in V$ and $r,s \in R$, we compute:

$$\begin{aligned} {[}E_{v,x}(r),E_{x,w}(s)] = E_{v,w}(rs) {\text { if }}v \ne w \end{aligned}$$

while for $v \ne y$ and $w \ne x$, $E_{v,w}(r)$ and $E_{x,y}(s)$ commute.

Write $E_{v,w} = E_{v,w} (1)$ for short. Let $E_V (R) \le {{\,\textrm{Aut}\,}}_R(R^V)$ be the group generated by all transvections $E_{v,w} (r)$, as (*v*, *w*) ranges over all pairs of distinct elements in *V* and *r* ranges over *R*.

##### Theorem 2.21

Suppose *V* is finite with $|V |\ge 3$. Then $E_V (\mathbb {Z}) \cong {{\,\textrm{SL}\,}}_{V} (\mathbb {Z})$ is generated by $S_V = \lbrace E_{v,w}:v,w \in V, v \ne w \rbrace$ and admits a presentation $\big \langle S_V \mid R_V \big \rangle$, where

$$\begin{aligned} R_V =&\big \lbrace [E_{v,x},E_{x,w}]E_{v,w} ^{-1} : v \ne w \big \rbrace \\&\cup \big \lbrace [E_{v,w},E_{x,y}] : v\ne y,w\ne x \big \rbrace \\&\cup \lbrace (E_{v_0 , w_0} E_{w_0 , v_0} ^{-1} E_{v_0 , w_0})^4 \rbrace \text {.} \end{aligned}$$

for any (fixed) distinct $v_0, w_0 \in V$.

Similarly, for *p* prime, $E_V (\mathbb {F}_p) \cong {{\,\textrm{SL}\,}}_{V} (\mathbb {F}_p)$ is generated by $S_V$, and is presented by $\big \langle S_V \mid R_V ^p \big \rangle$, where $R_V ^p = R_V \cup \lbrace E_{v_0,w_0} ^p \rbrace$.

##### Proof

This is covered in, for instance, Introduction to [9]. In this treatment, *V* is identified with $\lbrace 1,\ldots ,n\rbrace$ (for $n=|V|$) and $(v_0,w_0)$ is taken to be (1, 2). There is no loss of generality here, since all $E_{v,w}$ are conjugate in ${{\,\textrm{SL}\,}}_{V} (\mathbb {Z})$. $\square$

Now let $G = (V,E)$ be a finite connected graph. Recall that the *diameter* ${{\,\textrm{diam}\,}}(G)$ of *G* is the maximal distance between pairs of vertices in *G* in the path metric. Let $S_{G} = \lbrace E_{v,w},E_{w,v}: (v,w) \in E \rbrace \subseteq {{\,\textrm{SL}\,}}_V(\mathbb {Z})$.

##### Corollary 2.22

Let *G* and $S_G$ be as above. Then ${{\,\textrm{SL}\,}}_V(\mathbb {Z})$ admits a presentation $\big \langle S_G \mid R_G \big \rangle$ where $R_G \subseteq F(S_G)$ consists of words of length $O\big ( {{\,\textrm{diam}\,}}(G)^2 \big )$.

##### Proof

Since ${{\,\textrm{SL}\,}}_V(\mathbb {Z}) \cong \big \langle S_V \mid R_V \big \rangle$ as in Theorem 2.21, with $R_V \subseteq F(S_V)$ consisting of words of bounded length, it suffices to show that $S_V \subseteq B_{S_G} (M)$ for some $M = O \big ( {{\,\textrm{diam}\,}}(G)^2 \big )$. Let $v,w \in V$ be distinct, and let $p = (v=v_0,v_1,\ldots ,v_n=w)$ be a path of minimal length in *G* from *v* to *w*. We claim that, if $n \le 2^m$, then $E_{v,w} \in B_{S_G}(4^m)$. Certainly the claim holds for $m=0$. Assuming the claim for smaller *m*, let $x = v_{\lceil n/2 \rceil }$. Then *x* is at distance at most $2^{m-1}$ from both *v* and *w*, so $E_{v,x},E_{x,w} \in B_{S_G}(4^{m-1})$ by induction, and we have $E_{v,w} = [E_{v,x},E_{x,w}]$. The result then follows, since every pair *v*, *w* admits a path *p* of length $n \le {{\,\textrm{diam}\,}}(G)$. $\square$

## Symmetric enrichments

Let ${{\,\textrm{FSym}\,}}(\Omega )$ be the group of finitely supported permutations of the set $\Omega$. Note that

(i) $${{\,\textrm{FSym}\,}}(\Omega ) \vartriangleleft {{\,\textrm{Sym}\,}}(\Omega )$$;
(ii) For any finite subset $\Sigma \subseteq \Omega$, we have ${{\,\textrm{Sym}\,}}(\Sigma ) \le {{\,\textrm{FSym}\,}}(\Omega )$ (naturally identifying ${{\,\textrm{Sym}\,}}(\Sigma )$ with the set of permutations of $\Omega$ supported on $\Sigma$).

Let $\Gamma$ be a group acting transitively on the set $\Omega$. The action induces a homomorphism $\tilde{\phi }: \Gamma \rightarrow {{\,\textrm{Sym}\,}}(\Omega )$ which in turn induces $\phi : \Gamma \rightarrow {{\,\textrm{Aut}\,}}\big ( {{\,\textrm{FSym}\,}}(\Omega ) \big )$ ($\Gamma$ acts by conjugation, via $\tilde{\phi }$, on ${{\,\textrm{FSym}\,}}(\Omega )$).

### Definition 3.1

The *symmetric enrichment* of the action $\Omega \curvearrowleft \Gamma$ is the group $\mathcal {S}(\Gamma ,\Omega ) = {{\,\textrm{FSym}\,}}(\Omega ) \rtimes _{\phi } \Gamma$. If $\Omega = \Gamma$, equipped with the regular $\Gamma$-action, then we may write $\mathcal {S}(\Gamma )$ for $\mathcal {S}(\Gamma ,\Gamma )$.

### Remark 3.2

There is also ${{\,\textrm{FAlt}\,}}(\Omega ) \le {{\,\textrm{FSym}\,}}(\Omega )$, the index-2 subgroup consisting of finitely supported even permutations of $\Omega$. ${{\,\textrm{FAlt}\,}}(\Omega )$ is normalized by $\Gamma$, acting via $\phi$, so we may also define the *alternating enrichment* $\mathcal {A}(\Gamma ,\Omega ) = {{\,\textrm{FAlt}\,}}(\Omega )\rtimes _{\phi } \Gamma \le \mathcal {S}(\Gamma ,\Omega )$ and $\mathcal {A}(\Gamma ) \le \mathcal {S}(\Gamma )$.

Our main result on local embeddings of symmetric enrichments is the following.

### Theorem 3.3

Let $\Gamma$ be a LEF group, finitely generated by the symmetric set *S*, and let $\Omega \curvearrowleft \Gamma$ be a transitive LEF action. Then $\mathcal {S}(\Gamma ,\Omega )$ is finitely generated and LEF. Moreover, if $(\pi _n,\theta _n)$ is a sequence of local embeddings of $\big (B_S(n), B_{S,\omega _0}(n)\big )$ into $(Q_n,X_n)$ then

2 $$\begin{aligned} \gamma _{\Gamma ,\Omega }(n)! \preceq \mathcal {L}_{\mathcal {S}(\Gamma ,\Omega )} (n) \preceq |X_n |! |Q_n |\end{aligned}$$

In particular, for any finitely generated LEF group $\Gamma$:

3 $$\begin{aligned} \gamma _{\Gamma }(n)! \preceq \mathcal {L}_{\mathcal {S}(\Gamma )} (n) \preceq \mathcal {L}_{\Gamma } (n)! \end{aligned}$$

### Corollary 3.4

If $\Omega$ is infinite then $\mathcal {L}_{\mathcal {S}(\Gamma ,\Omega )}(n) \succeq n!$. In particular $\mathcal {S}(\Gamma ,\Omega )$ is not ELEF.

### Proof

This follows from (2) and the fact that every infinite Schreier graph of a finitely generated group has growth at least linear and at most exponential. $\square$

### Corollary 3.5

If $\Omega$ is infinite and $\Omega \curvearrowleft \Gamma$ is a transitive LEF action, then $\mathcal {S}(\Gamma ,\Omega )$ is not finitely presentable.

### Proof

By Theorem 3.3 and Proposition 2.13, it suffices to prove that $\mathcal {S}(\Gamma ,\Omega )$ is not residually finite. This is so because $\mathcal {S}(\Gamma ,\Omega )$ contains ${{\,\textrm{FAlt}\,}}(\Omega )$, an infinite simple group. $\square$

We imagine that the conclusion of Corollary 3.5 holds under much weaker conditions on the action, and may be known to some experts (in a related vein, see the criteria for finite presentability of a wreath product in [10]). We are, however, not aware of an existing reference.

### Example 3.6

If $k \ge 1$ and $\Gamma = \mathbb {Z}^k$ then by Example 2.18 (i) $\mathcal {L}_{\Gamma }(n) \approx \gamma _{\Gamma } (n) \approx n^k$, so by (3), $\mathcal {L}_{\mathcal {S}(\Gamma )} (n) \approx (n^k)!$.

### Example 3.7

For $k \ge 2$, if either $\Gamma = F_k$ or $\Gamma = {{\,\textrm{SL}\,}}_k(\mathbb {Z})$ then by Example 2.18 (iii) and (iv) $\mathcal {L}_{\Gamma }(n) \approx \gamma _{\Gamma } (n) \approx \exp (n)$, so by (3), $\mathcal {L}_{\mathcal {S}(\Gamma )} (n) \approx (\exp (n))!$.

We now prove Theorem 3.3. The first step is to show finite generation of $\mathcal {S}(\Gamma ,\Omega )$.

### Lemma 3.8

Let *S* be a symmetric generating set for $\Gamma$ and let $\omega _0\in \Omega$. Let $G_n = (V_n,E_n)$ be the finite graph with vertex set $V_n =B_{S,\omega _0}(n)$ and edge set $E_n=\lbrace (\omega _0 g,\omega _0 sg):s\in S,g\in B_S (n-1)\rbrace$. Then $G_n$ is a connected graph, and contains a maximal tree $T_n$ of diameter at most 2*n*.

### Proof

We construct $T_n$ by induction on *n*. The base case is $T_1$, which is the star graph with leaves $\omega _0 S {\setminus } \lbrace \omega _0 \rbrace$. For $n \ge 2$, and given $T_{n-1}$, we choose for all $v \in V_n \setminus V_{n-1}$ an *n*-tuple $(s_1, \ldots , s_n)$ such that $v = \omega _0 s_1 \cdots s_n$. Writing $s_v = s_1$, $g_v = s_2 \cdots s_n$, we have $(\omega _0 g_v,v)=(\omega _0 g_v,\omega _0 s_v g_v) \in E_n$ and $\omega _0 g_v \in V_{n-1}$. Define the graph $T_n$ on $V_n$ by $E(T_n) = E(T_{n-1}) \cup \lbrace (\omega _0 g_v,v): v \in V_n {\setminus } V_{n-1} \rbrace$. Then $T_n$ is a tree on $V_n$ (since $T_{n-1}$ is a tree on $V_{n-1}$ and the elements of $V_n \setminus V_{n-1}$ are leaves of $T_n$). Moreover, every element of $V_n \setminus V_{n-1}$ is at distance 1 in $T_n$ from an element of $V_{n-1}$, so ${{\,\textrm{diam}\,}}(T_n) \le {{\,\textrm{diam}\,}}(T_{n-1}) + 2 \le 2n$. Since $G_n$ contains $T_n$ as a subgraph on the same vertex set, $G_n$ is connected and $T_n$ is a maximal tree. $\square$

### Lemma 3.9

If $\Gamma$ is finitely generated by the symmetric set *S* then for any $\omega _0 \in \Omega$, $\mathcal {S}(\Gamma ,\Omega )$ is finitely generated by $S \cup T_{S,\omega _0}$, where

$$\begin{aligned} T_{S,\omega _0} = \lbrace (\omega _0 \;\omega _0 s) : s \in S \rbrace \subseteq {{\,\textrm{FSym}\,}}(\Gamma ). \end{aligned}$$

### Proof

Let $\sigma \in {{\,\textrm{FSym}\,}}(\Omega )$ and $g \in \Gamma$. Certainly $g \in \langle S \rangle$. By transitivity of $\Omega \curvearrowleft \Gamma$, there exists $n \in \mathbb {N}$ such that ${{\,\textrm{supp}\,}}(\sigma ) \subseteq {{\,\textrm{Sym}\,}}\big ( B_{S,\omega _0}(n) \big )$. Let $T_n$ be as in Lemma 3.8. By Remark 2.20, ${{\,\textrm{Sym}\,}}\big ( B_{S,\omega _0}(n) \big )$ is generated by $S_{T_n} = \lbrace s_e: e \in E(T_n) \rbrace$ as in the statement of Theorem 2.19. For $e \in E(T_n)$, there exist $h \in \Gamma$ and $s \in S$ such that $s_e = (\omega _0 h \; \omega _0 sh) = (\omega _0 \; \omega _0 s)^h \in \langle S \cup T_{S,\omega _0} \rangle$. $\square$

The upper bound in (2) will follow from the next construction.

### Lemma 3.10

Let $\Gamma$, *N* be groups, let $\phi : \Gamma \rightarrow {{\,\textrm{Aut}\,}}(N)$ be a homomorphism, and let $S \subseteq \Gamma$, $T \subseteq N$ be symmetric subsets. Then within $N \rtimes _{\phi } \Gamma$,

4 $$\begin{aligned} B_{S \cup T} (n) \subseteq B_{C(S,T,n)} (n) \cdot B_S (n) \end{aligned}$$

where $$C(S,T,n) = \lbrace t^w: w \in B_S (n-1),t\in T \rbrace \subseteq N$$.

### Proof

For $g \in B_{S \cup T} (n)$, there exist $s_1, \ldots , s_n \in S \cup \lbrace 1 \rbrace$ and $t_1, \ldots , t_n \in T \cup \lbrace 1 \rbrace$ such that $g = t_1 s_1 \cdots t_n s_n = h\cdot (s_1 \cdots s_n)$, where

$$\begin{aligned} h = t_1 \big ( t_2 ^{s_1 ^{-1}} \big ) \big ( t_3 ^{(s_1 s_2) ^{-1}} \big ) \cdots \big ( t_n ^{(s_1 \cdots s_{n-1}) ^{-1}} \big ) \end{aligned}$$

and for $$1 \le k \le n$$, $$t_k ^{(s_1 \cdots s_{k-1})^{-1}} \in C(S,T,n)$$. $$\square $$

### Proposition 3.11

Let $I(n)={{\,\textrm{Sym}\,}}\big ( B_{S,\omega _0}(n)\big ) \cdot B_S (n) \subseteq \mathcal {S}(\Gamma ,\Omega )$ and let $(\pi _n,\theta _n)$ be a local embedding of $\big (B_S(n), B_{S,\omega _0}(n)\big )$ into $(Q_n,X_n)$. Then there is a local embedding of *I*(*n*) into $\mathcal {S}(Q_n,X_n)$.

### Proof

Write $B(n) = B_{S,\omega _0}(n)$. Let $\tilde{\theta }_n: {{\,\textrm{Sym}\,}}\big ( B(n)\big ) \rightarrow {{\,\textrm{Sym}\,}}(X_n)$ be the (injective) homomorphism induced by the injection $\theta _n: B(n) \rightarrow X_n$; that is,

$$\begin{aligned} {[}\theta _n (\omega )]\tilde{\theta }_n (\sigma ) = \theta _n (\omega \sigma ){\text { and }}[x]\tilde{\theta }_n (\sigma ) = x{\text { for }}x \in X_n \setminus {{\,\textrm{im}\,}}(\theta _n). \end{aligned}$$

Let $\Phi _n: I(n) \rightarrow \mathcal {S}(Q_n,X_n)$ be given by $\Phi _n (\sigma \cdot g)=\tilde{\theta }_n (\sigma ) \cdot \pi _n (g)$. $\Phi _n$ is clearly injective; we claim that it is a local embedding.

For $\sigma _1,\sigma _2 \in {{\,\textrm{Sym}\,}}\big ( B(n)\big )$, $g_1, g_2 \in B_S (n)$, we have $(\sigma _1 g_1) (\sigma _2 g_2) = \sigma _1 \sigma _2 ^{g_1 ^{-1}} g_1 g_2 \in I(n)$ iff (i) $g_1 g_2 \in B_S (n)$ and (ii) ${{\,\textrm{supp}\,}}(\sigma _1 \sigma _2 ^{g_1 ^{-1}}) \subseteq B(n)$. Since ${{\,\textrm{supp}\,}}(\sigma _1) \subseteq B(n)$, (ii) is equivalent to (ii’) ${{\,\textrm{supp}\,}}(\sigma _2) \subseteq B(n) g_1 \cap B(n)$. Under these conditions,

$$\begin{aligned} \Phi _n \big ( (\sigma _1 g_1) (\sigma _2 g_2) \big )&= \Phi _n \big ( \sigma _1 \sigma _2 ^{g_1 ^{-1}} g_1 g_2 \big ) \\&= \tilde{\theta }_n \big ( \sigma _1 \sigma _2 ^{g_1 ^{-1}} \big ) \pi _n (g_1 g_2) \\&= \tilde{\theta }_n (\sigma _1) \tilde{\theta }_n (\sigma _2 ^{g_1 ^{-1}}) \pi _n (g_1)\pi _n (g_2) \text { (by (i))} \end{aligned}$$

while

$$\begin{aligned} \Phi _n (\sigma _1 g_1)\Phi _n (\sigma _2 g_2)&= \tilde{\theta }_n (\sigma _1) \pi _n (g_1) \tilde{\theta }_n (\sigma _2) \pi _n (g_2) \\&= \tilde{\theta }_n (\sigma _1) \tilde{\theta }_n (\sigma _2)^{\pi _n (g_1)^{-1}} \pi _n (g_1)\pi _n (g_2) \end{aligned}$$

so, writing $\tau _1 = \tilde{\theta }_n (\sigma _2)^{\pi _n (g_1)^{-1}}, \tau _2 = \tilde{\theta }_n (\sigma _2 ^{g_1 ^{-1}}) \in {{\,\textrm{Sym}\,}}(X_n)$, it suffices to check that $\tau _1 = \tau _2$. Since, by (ii’), ${{\,\textrm{supp}\,}}(\sigma _2) \subseteq B(n) g_1 \cap B(n)$,

$$\begin{aligned} {{\,\textrm{supp}\,}}(\tilde{\theta }_n (\sigma _2)) \subseteq \theta _n (B(n)) \pi _n (g_1) \cap \theta _n (B(n));\, {{\,\textrm{supp}\,}}(g_1 \sigma _2 g_1 ^{-1}) \subseteq B(n) g_1 ^{-1} \cap B(n) \end{aligned}$$

so that

5 $$\begin{aligned} {{\,\textrm{supp}\,}}( \tau _1 ) \subseteq \theta _n (B(n))\pi _n (g_1)^{-1} \cap \theta _n (B(n)) \end{aligned}$$

and

6 $$\begin{aligned} {{\,\textrm{supp}\,}}(\tau _2)&\subseteq \theta _n (B(n)g_1 ^{-1} \cap B(n)) \end{aligned}$$

7 $$\begin{aligned}&\subseteq \theta _n (B(n))\pi _n (g_1)^{-1} \cap \theta _n (B(n)) \end{aligned}$$

We verify that for all $x \in X_n$, $(x)\tau _1 = (x)\tau _2$. First suppose $x\in \theta _n (B(n)g_1 ^{-1}\cap B(n))$, so that there are $\omega , \tilde{\omega } \in B(n)$ with $\omega = \tilde{\omega } g_1 ^{-1}$ are such that $x = \theta _n (\omega ) = \theta _n (\tilde{\omega } g_1 ^{-1})$ so $x = \theta _n (\tilde{\omega }) \pi _n (g_1 ^{-1})$. Thus,

$$\begin{aligned} \tau _1 : x \mapsto \theta _n (\tilde{\omega }) \tilde{\theta }_n (\sigma _2) \pi _n (g_1)^{-1} \end{aligned}$$

while

$$\begin{aligned} \tau _2 :x \mapsto \theta _n (\tilde{\omega } \sigma _2 g_1 ^{-1}) = \theta _n (\tilde{\omega } \sigma _2) \pi _n (g_1 ^{-1}) =\theta _n(\tilde{\omega })\tilde{\theta }_n(\sigma _2)\pi _n (g_1 ^{-1}) \end{aligned}$$

(as ${{\,\textrm{supp}\,}}(\sigma _2) \subseteq B(n)g_1 \cap B(n)$, so either $\tilde{\omega } \notin {{\,\textrm{supp}\,}}(\sigma _2)$ or $\tilde{\omega }\sigma _2,\tilde{\omega }\sigma _2g_1 ^{-1}\in B(n)$).

Next suppose $x \in X_n \setminus \theta _n (B(n))$. Then by (5) and (7), $\tau _1$ and $\tau _2$ fix *x*. Finally suppose $x\in \theta _n(B(n)){\setminus }\theta _n(B(n)g_1 ^{-1})$. Then by (6) $\tau _2$ fixes *x*. If in addition $x \notin \theta _n (B(n)) \pi _n (g_1)^{-1}$, then by (5) $\tau _1$ fixes *x* as well. Therefore, suppose that there exist $\omega ,\tilde{\omega } \in B(n)$ such that $x =\theta _n (\omega ) =\theta _n (\tilde{\omega })\pi _n(g_1)^{-1}$. Then $\tilde{\omega } g_1 ^{-1} \notin B(n)$ (else $\theta _n (\tilde{\omega } g_1 ^{-1}) =\theta _n (\tilde{\omega })\pi _n(g_1)^{-1} =x=\theta _n (\omega )$, so $\omega = \tilde{\omega }g_1 ^{-1}$ and $x \in \theta _n (B(n)g_1 ^{-1})$, a contradiction). Thus, $\tilde{\omega } \notin B(n)g_1 \supseteq {{\,\textrm{supp}\,}}(\sigma _2)$, so

$$\begin{aligned} \tau _1 : x = \theta _n (\tilde{\omega })\pi _n (g_1)^{-1}&\mapsto \theta _n (\tilde{\omega })\tilde{\theta }_n (\sigma _2)\pi _n (g_1)^{-1} \\&= \theta _n (\tilde{\omega }\sigma _2) \pi _n (g_1)^{-1} \\&= \theta _n (\tilde{\omega }) \pi _n (g_1)^{-1} \\&= x \end{aligned}$$

$$\square $$

### Proof of Theorem 3.3

Let *I*(*n*) be as in Proposition 3.11. To prove the upper bound in (2), it suffices to show that $B_{S \cup T_{S,\omega _0}} (n) \subseteq I(n)$. Taking $T = T_{S,\omega _0}$ in Lemma 3.10, it suffices to show that for $t \in T_{S,\omega _0}$ and $w \in B_S (n-1)$, $t^w \in {{\,\textrm{Sym}\,}}\big ( B_{S,\omega _0}(n) \big )$. This is so, since letting $s \in S$ be such that $t = (\omega _0 \; \omega _0 s)$, so that ${{\,\textrm{supp}\,}}\big ( t ^{w} \big ) = \lbrace \omega _0 w,\omega _0 s w \rbrace \subseteq B_{S,\omega _0}(n)$.

For the lower bound, we apply Proposition 2.15, replacing $\Gamma$ by $\mathcal {S}(\Gamma ,\Omega )$ and *S* by $S \cup T_{S,\omega _0}$ as in Lemma 3.9, and taking $H_n = {{\,\textrm{Sym}\,}}\big ( B_{S,\omega _0}(n)\big )$. Let the graph $G_n$ and the tree $T_n$ be as in Lemma 3.8. Let $H_n = \big \langle S_{T_n} \mid R_{T_n} \big \rangle$ be as in Theorem 2.19, and set $S^{\prime \prime } _n = S^{\prime } _n = S_{T_n}$. By the description of $R_{T_n}$ from Theorem 2.19, we can take $g(n) = 8$ for all *n*. Further, by the definition of the graph $G_n$ from Lemma 3.8 and the generating set $S_{T_n}$ from Theorem 2.19, for any $s^{\prime \prime } \in S^{\prime \prime } _n = S_{T_n}$, there exist $h \in B_S (n-1)$ and $s \in S$ such that

$$\begin{aligned} s^{\prime \prime } = (\omega _0 h \; \omega _0 sh) = (\omega _0 \; \omega _0 s)^h \in B_{S \cup T_{S,\omega _0}} (2n-1). \end{aligned}$$

We may therefore take $f(n) = 2n-1$ in Proposition 2.15 and obtain that for all *n*,

$$\begin{aligned} \mathcal {L}_{\mathcal {S}(\Gamma ,\Omega )} ^{S\cup T_{S,\omega _0}}(16n-8) \ge \mathcal {R}_{{{\,\textrm{Sym}\,}}( B_{S,\omega _0}(n))} ^{S_{T_n}} (8). \end{aligned}$$

The right-hand side of the last inequality is the order of a quotient of ${{\,\textrm{Sym}\,}}\big ( B_{S,\omega _0}(n)\big )$, into which $B_{S_{T_n}} (8)$ injects. For $n \ge 5$, $|B_{S,\omega _0}(n) |\ge 5$ so ${{\,\textrm{Sym}\,}}\big ( B_{S,\omega _0}(n)\big )$ has no proper quotients of order greater than 2, while $|B_{S_{T_n}} (8)|\ge 8$, so

$$\begin{aligned} \mathcal {R}_{{{\,\textrm{Sym}\,}}( B_{S,\omega _0}(n))} ^{S_{T_n}} (8) = \big |{{\,\textrm{Sym}\,}}\big ( B_{S,\omega _0}(n)\big ) \big |= \gamma _{\Gamma ,\Omega } ^S (n)!. \end{aligned}$$

$$\square $$

### Remark 3.12

(i) The conclusions of Theorem 3.3 are also valid for the alternating enrichment $\mathcal {A}(\Gamma ,\Omega )$. The upper bounds hold simply because $\mathcal {A}(\Gamma ,\Omega ) \le \mathcal {S}(\Gamma ,\Omega )$. The lower bounds follow by applying Proposition 2.15 to the presentations of the alternating groups, induced from those appearing in Theorem 2.19 for the symmetric groups by the Reidemeister–Schreier procedure.
(ii) For $\Omega \curvearrowleft \Gamma$ we have an induced action $\Omega \curvearrowleft \mathcal {S}(\Gamma ,\Omega )$. A straightforward extension of the proof of Proposition 3.11 shows that if the former action is LEF then so is the latter.

## Elementary enrichments

Let $E_{v,w}(r)$, $E_{v,w} = E_{v,w}(1)$ and $E_V (R)$ be as in 2.4.2. For $W \subseteq V$, there is a natural embedding $E_W (R) \le E_V (R)$, induced by acting as the identity on all basis vectors in $V {\setminus } W$. If *X* is the ascending union of finite subsets $V_n \subseteq V$, then

$$\begin{aligned} E_V (R) = \cup _n E_{V_n} (R) \end{aligned}$$

Now let $\Gamma$ be a group, acting transitively on the set *V*. $\Gamma$ acts by automorphisms on $E_V (R)$ via:

$$\begin{aligned} (E_{v,w}(r))^g = E_{vg,wg}(r) \end{aligned}$$

We may therefore define the *R-elementary enrichment* of the action $V \curvearrowleft \Gamma$ over *R* to be the group:

$$\begin{aligned} \mathcal {E}(\Gamma ,V,R) = E_V (R) \rtimes \Gamma . \end{aligned}$$

In case $V = \Gamma$ and the action is regular, we write $\mathcal {E} (\Gamma ,R)$ for short.

Passing from $\Gamma$ to $\mathcal {E}(\Gamma ,V,R)$ does not typically preserve residual finiteness.

### Proposition 4.1

Suppose $\Gamma$ is finitely generated and *V* is infinite. Then $\mathcal {E}(\Gamma ,V,R)$ is not residually finite.

We imagine the conclusion of Proposition 4.1 to be known to experts but, being unable to locate a proof in the literature, we include one here.

### Lemma 4.2

Suppose $|V |\ge 6$. Let $N \vartriangleleft \mathcal {E}(\Gamma ,V,R)$. Suppose there exist $v \ne w$ such that $E_{v,w} \in N$. Then $E_V (R) \le N$.

### Proof

Let $x,y \in V$ be distinct and let $r \in R$. Claim that $E_{x,y} (r) \in N$. Let $t,u \in V {\setminus } \lbrace v,w,x,y \rbrace$ be distinct. Then,

$$\begin{aligned} E_{t,u}(r) = \big [ E_{t,v}(r) ,[E_{v,w},E_{w,u}] \big ] \in N \end{aligned}$$

so $$E_{x,y}(r) = \big [ E_{x,t},[E_{t,u}(r),E_{u,y}] \big ] \in N$$. $$\square $$

### Proof of Proposition 4.1

Let $N \vartriangleleft _f \mathcal {E}(\Gamma ,V,R)$. We claim that $E_V (R) \le N$, from which the conclusion follows. By Lemma 4.2, it suffices to show that there exist distinct $v,w \in V$ such that $E_{v,w} \in N$.

If there exists $v \in V$ and $g \in N \cap \Gamma$ such that $v,v^g$ $v^{g^2}$ are distinct, then

$$\begin{aligned} 1 = [E_{v,v^g}, E_{v,v^g}]&\equiv [E_{v,v^g}, g^{-1}E_{v,v^g} g] \mod N \\&= [E_{v,v^g},E_{v^g,v^{g^2}}] \\&= E_{v,v^{g^2}} \end{aligned}$$

so $E_{v,v^{g^2}} \in N$, as required. Thus, we may assume that for all $v \in V$ and $g \in N \cap \Gamma$, $v^{g^2} = v$. It follows that, where $\rho : \Gamma \rightarrow {{\,\textrm{Sym}\,}}(V)$ is the homomorphism induced by $V \curvearrowleft \Gamma$, $\rho (N \cap \Gamma )$ is of exponent at most 2 and hence is abelian. Moreover, $\rho (N \cap \Gamma )$ is finitely generated, (since $N \cap \Gamma \le _f \Gamma$ is), so $\rho (N \cap \Gamma )$ is a finite group. This is a contradiction, since by transitivity of $V \curvearrowleft \Gamma$, $\rho (\Gamma )$ is infinite, hence so is $\rho (N \cap \Gamma ) \le _f \rho (\Gamma )$. $\square$

By contrast $\mathcal {E}(\Gamma ,\Omega ,R)$ *can* admit many local embeddings. Here we emphasize the cases $R=\mathbb {Z}$ and $\mathbb {F}_p$. First we must deal with the issue of finite generation.

### Proposition 4.3

Let $\Gamma$ be finitely generated by the symmetric set *S* and let $\Omega \curvearrowleft \Gamma$ be a transitive action. Let:

$$\begin{aligned} T(\omega _0) = \lbrace E_{\omega _0,\omega _0 s} : s \in S \rbrace \cup \lbrace E_{\omega _0 s,\omega _0} : s \in S \rbrace \subseteq E_{\Omega } (R). \end{aligned}$$

Then for all $\omega , \psi \in \Omega$ distinct, $E_{\omega , \psi } \in \langle S \cup T(\omega _0) \rangle$. Moreover, there exists an absolute constant $C>0$ such that for all $n \in \mathbb {N}$ and $g \in \Gamma$, if $|g |_S \le n$ then

8 $$\begin{aligned} \big |E_{\omega _0 , \omega _0 g} \big |_{S \cup T(\omega _0)} , \big |E_{\omega _0 g, \omega _0} \big |_{S \cup T(\omega _0)} \le Cn^2 . \end{aligned}$$

### Proof

We can reduce to the case $\omega = \omega _0$ or $\psi = \omega _0$, since otherwise:

$$\begin{aligned} E_{\omega , \psi } = \big [ E_{\omega , \omega _0} , E_{\omega _0 , \psi } \big ]. \end{aligned}$$

Further, since $\Omega \curvearrowleft \Gamma$ is transitive, we may write $\omega = \omega _0 g$ and $\psi = \omega _0 h$ for some $g,h \in \Gamma$. Thus, if $\omega \ne \omega _0$ we have:

$$\begin{aligned} E_{\omega ,\omega _0}=E_{\omega _0 g ,\omega _0}=(E_{\omega _0 ,\omega _0 g^{-1}} )^g \end{aligned}$$

so it suffices to prove (8). To this end, we prove, by induction on $m \in \mathbb {N}$, that

9 $$\begin{aligned} L_m = \max _{|g |_S \le 2^m , \omega _0 g \ne \omega _0}|E_{\omega _0 , \omega _0 g} |_{S \cup T(\omega _0)} \le (C/2) 4^m . \end{aligned}$$

The corresponding bound on $|E_{\omega _0 g, \omega _0} |_{S \cup T(\omega _0)}$ follows by symmetry and the inequality (8) for general *n* follows since $2^m \le n \le 2^{m+1}$ for some *m*. To this end, take $g \in B_S (2^m)$ with $\omega _0 g \ne \omega _0$. If there exists $s \in S$ such that $\omega _0 g = \omega _0 s$, then $E_{\omega _0, \omega _0 g} \in T(\omega _0)$. This covers the case $m=0$. Assume therefore that this is not the case. Then there exist $h, k \in \Gamma$ with $|h |_S, |k |_S \le 2^{m-1}$ such that $g = hk$ and $\omega _0$, $\omega _0 k$, and $\omega _0 g$ are all distinct (so that $\omega _0 \ne \omega _0 h$ as well). We have:

$$\begin{aligned} E_{\omega _0 , \omega _0 g} = \big [ E_{\omega _0 , \omega _0 k} , E_{\omega _0 k , \omega _0 g} \big ] = \big [ E_{\omega _0 , \omega _0 k} , (E_{\omega _0, \omega _0 h})^k \big ] \end{aligned}$$

so that

$$\begin{aligned} |E_{\omega _0 , \omega _0 g} |_{S \cup T(\omega _0)}&\le 2 |E_{\omega _0 , \omega _0 h} |_{S \cup T(\omega _0)} + 2 |E_{\omega _0 , \omega _0 k} |_{S \cup T(\omega _0)} + 4 |k |_S \\&\le 4 L_{m-1} + 2^{m+1} \end{aligned}$$

Taking the maximum over all such *g*, $L_m \le 4 L_{m-1} + 2^{m+1}$, and solving the corresponding recurrence yields (9). $\square$

### Corollary 4.4

Let $R=\mathbb {Z}$ or $\mathbb {F}_p$. Let $\Gamma$, *S*, $\Omega$ and $T(\omega _0)$ be as in Proposition 4.3. Then $\mathcal {E}(\Gamma ,\Omega ,R)$ is finitely generated by $S \cup T(\omega _0)$.

### Proof

$\mathcal {E}(\Gamma ,\Omega ,R)$ is generated by $\Gamma$ and $E_{\Omega } (R)$. $\Gamma$ is generated by *S*, and by Proposition 4.3, $E_{\Omega } (R) \subseteq \langle S \cup T(\omega _0) \rangle$. $\square$

Now we can bound the LEF growth of $\mathcal {E}(\Gamma ,\Omega ,\mathbb {Z})$ and $\mathcal {E}(\Gamma ,\Omega ,\mathbb {F}_p)$. Let $T(\omega _0)$ be as in Proposition 4.3.

### Theorem 4.5

Let $\Gamma$ be a LEF group, finitely generated by *S*, and let $\Omega \curvearrowleft \Gamma$ be a transitive LEF action. Then $\mathcal {E}(\Gamma ,\Omega ,\mathbb {Z})$ and $\mathcal {E}(\Gamma ,\Omega ,\mathbb {F}_p)$ are LEF. Moreover, if $(\pi _n,\theta _n)$ is a sequence of local embeddings of $\big (B_S(n), B_{S,\omega _0}(n)\big )$ into $(Q_n,X_n)$ then there exists $C > 0$ such that

10 $$\begin{aligned} \exp \big (n \gamma _{\Gamma ,\Omega } ^{S,\omega _0}(n^{1/2})^2 / C \big ) \le \mathcal {L}_{\mathcal {E}(\Gamma ,\Omega ,\mathbb {Z})} ^{S\cup T(\omega _0)} (n) \le \exp \big ( Cn |X_n |^2 \big ) |Q_n |\text {.} \end{aligned}$$

11 $$\begin{aligned} \exp \big (\gamma _{\Gamma ,\Omega } ^{S,\omega _0}(n^{1/2})^2 /C \big ) \le \mathcal {L}_{\mathcal {E}(\Gamma ,\Omega ,\mathbb {F}_p)} ^{S\cup T(\omega _0)} (n) \le \exp \big ( C|X_n |^2 \big ) |Q_n |\text {.} \end{aligned}$$

In particular, for any finitely generated LEF group $\Gamma$,

12 $$\begin{aligned} \exp \big (n \gamma _{\Gamma } (n^{1/2})^2 \big ) \preceq \mathcal {L}_{\mathcal {E}(\Gamma ,\mathbb {Z})} (n) \preceq \exp \big ( n \mathcal {L}_{\Gamma }(n)^2 \big )\text {.} \end{aligned}$$

13 $$\begin{aligned} \exp \big (\gamma _{\Gamma } (n^{1/2})^2/C \big ) \preceq \mathcal {L}_{\mathcal {E}(\Gamma ,\mathbb {F}_p)} (n) \preceq \exp \big (C \mathcal {L}_{\Gamma }(n)^2 \big )\text {.} \end{aligned}$$

The next observation follows from Theorem 4.5 much as Corollary 3.4 follows from Theorem 3.3.

### Corollary 4.6

If $\Omega$ is infinite, then $\mathcal {E}(\Gamma ,\Omega ,\mathbb {Z})$ is not *ELEF*.

### Corollary 4.7

If $\Gamma$ is a LEF group and $\Omega$ is infinite, then $\mathcal {E}(\Gamma ,\Omega ,\mathbb {Z})$ and $\mathcal {E}(\Gamma ,\Omega ,\mathbb {F}_p)$ are not finitely presentable.

### Proof

Let $R = \mathbb {Z}$ or $\mathbb {F}_p$. If $\Gamma$ is finitely generated, then by Proposition 4.1 and Theorem 4.5, $\mathcal {E}(\Gamma ,\Omega ,R)$ is LEF but not residually finite, so by Proposition 2.13 is not finitely presentable. If $\Gamma$ is *not* finitely generated, then neither is $\mathcal {E}(\Gamma ,\Omega ,R)$ (since the former is a quotient of the latter). $\square$

For the lower bound in (10), we shall need the congruence subgroup property for ${{\,\textrm{SL}\,}}_V (\mathbb {Z})$ with $3 \le |V |< \infty$. For $q \in \mathbb {N}$ let $\pi _q: {{\,\textrm{SL}\,}}_V(\mathbb {Z}) \rightarrow {{\,\textrm{SL}\,}}_V(\mathbb {Z}/q\mathbb {Z})$ be the *congruence homomorphism* (given, for $A \in {{\,\textrm{SL}\,}}_V(\mathbb {Z})$, by $\pi _q (A)_{v,w} = A_{v,w} \mod q$). If $r \in \mathbb {N}$ with $q \mid r$, then we also denote by $\pi _q$ the map ${{\,\textrm{SL}\,}}_V(\mathbb {Z}/r\mathbb {Z}) \rightarrow {{\,\textrm{SL}\,}}_V(\mathbb {Z}/q\mathbb {Z})$ to which the former descends. The following result is proved in [2].

### Theorem 4.8

Let $3 \le |V |< \infty$, let *Q* be a finite group, and let $\phi : {{\,\textrm{SL}\,}}_V(\mathbb {Z}) \rightarrow Q$ be a homomorphism. Then there exists $q \in \mathbb {N}$ and a homomorphism $\psi : {{\,\textrm{SL}\,}}_V(\mathbb {Z}/q\mathbb {Z}) \rightarrow Q$ such that $\phi = \psi \circ \pi _q$.

The *mod*-*q* *principal congruence subgroup* of ${{\,\textrm{SL}\,}}_V(\mathbb {Z})$ is:

$$\begin{aligned} K_V (q) = \ker \big ( \pi _q : {{\,\textrm{SL}\,}}_V(\mathbb {Z}) \rightarrow {{\,\textrm{SL}\,}}_V(\mathbb {Z}/q\mathbb {Z}) \big ) = {{\,\textrm{SL}\,}}_V(\mathbb {Z}) \cap \big ( I_V + q \cdot \textbf{M}_V (\mathbb {Z}) \big ) \end{aligned}$$

where $\textbf{M}_V (\mathbb {Z})$ denotes the set of integer square matrices whose rows and columns are both indexed by *V*. This includes the case $K_V(1) = {{\,\textrm{SL}\,}}_V (\mathbb {Z})$. We supplement the congruence subgroup property with some familiar facts about the normal subgroup structure of ${{\,\textrm{SL}\,}}_V (\mathbb {Z})$.

### Lemma 4.9

(Chinese Remainder Theorem) Let *V* be finite. Let $q_1$, $q_2$ be coprime integers. Then $(\pi _{q_1} \times \pi _{q_2}): {{\,\textrm{SL}\,}}_V (\mathbb {Z} / q_1 q_2 \mathbb {Z}) \rightarrow {{\,\textrm{SL}\,}}_V (\mathbb {Z} / q_1 \mathbb {Z}) \times {{\,\textrm{SL}\,}}_V (\mathbb {Z} / q_2 \mathbb {Z})$ is an isomorphism. Moreover, for $q^{\prime } \mid q_1$, the preimage of $\pi _{q_1} \big (K_V (q^{\prime })\big ) \times 1$ under this isomorphism is $\pi _q \big ( K_V (q^{\prime } q_2)\big )$.

### Proposition 4.10

Let *V* be finite with $|V |\ge 3$. Suppose that $q \ge 4$ is composite, and that *p* is a prime divisor of *q*. Let $N \vartriangleleft {{\,\textrm{SL}\,}}_V (\mathbb {Z})$ with $K_V (q) \le N$. Suppose there exists $W \subseteq V$ with $|W |\ge 2$, such that $K_W (q/p) \le N$. Then $K_V (q/p) \le N$.

### Proof

We distinguish two cases. If $p^2$ does not divide *q*, then by Lemma 4.9 we have that $K_V (q/p)/K_V (q) \cong {{\,\textrm{SL}\,}}_V (\mathbb {Z}/p\mathbb {Z})$ is quasisimple (by the assumption on $|V|$), with:

$$\begin{aligned} K_W (q/p) K_V (q) / K_V (q) \le N \cap K_V (q/p) / K_V (q) \vartriangleleft K_V (q/p)/K_V (q), \end{aligned}$$

so $N \cap K_V (q/p) / K_V (q)$ is not central in $K_V (q/p)/K_V (q)$. We conclude:

$$\begin{aligned} N \cap K_V (q/p) / K_V (q) = K_V (q/p)/K_V (q) \end{aligned}$$

so that $$K_V (q/p) \subseteq N$$.

Alternatively, if $p^2$ divides *q*, then $K_V (q/p) / K_V (q) \cong (\mathbb {Z}/p\mathbb {Z}) ^ {|V |^2 - 1}$ is spanned by $\lbrace E_{v,w}(q/p):v \ne w \rbrace \cup \lbrace D_{v,w}(1 + q/p):v\ne w \rbrace$ where for $\alpha \in (\mathbb {Z}/q\mathbb {Z})^{*}$, $D_{v,w} (\alpha )$ acts on the basis *V* via:

$$\begin{aligned} D_{v,w} (\alpha ) : v \mapsto \alpha v,w \mapsto \alpha ^{-1} w, u \mapsto u{\text { for }}v \ne u \ne w. \end{aligned}$$

For any $v,w,v',w' \in V$ with $v\ne w$, $v'\ne w'$, $E_{v,w}(q/p)$ (respectively, $D_{v,w}(1 + q/p)$) is conjugate to $E_{v',w'}(q/p)^{\pm 1}$ (resp. $D_{v',w'}(1 + q/p)^{\pm 1}$) via a permutation matrix. Since we have $E_{v,w}(q/p), D_{v,w}(1 + q/p) \in N / K_V(q)$, for some $v,w \in W$, the required claim follows. $\square$

### Proposition 4.11

Let *V* be finite with $|V |\ge 3$ and suppose $(|V |,q)=1$. Let $K_V (q) \le N \vartriangleleft {{\,\textrm{SL}\,}}_V(\mathbb {Z})$.

(i) If $\pi _q (N)$ is central in ${{\,\textrm{SL}\,}}_V(\mathbb {Z}/q\mathbb {Z})$, then $|N: K_V (q) |\le |V |^{\log _2 q}$;
(ii) If $\pi _q (N)$ is non-central in ${{\,\textrm{SL}\,}}_V(\mathbb {Z}/q\mathbb {Z})$, then *q* has a proper divisor *r* such that $K_V (r) \le N$.

### Proof

Let $q = p_1 ^{a_1} \cdots p_l ^{a_l}$ be the prime factorization of *q*. By Lemma 4.9,

$$\begin{aligned} {{\,\textrm{SL}\,}}_V(\mathbb {Z}/q\mathbb {Z}) \cong \prod _{i=1} ^l {{\,\textrm{SL}\,}}_V(\mathbb {Z}/p_i ^{a_i} \mathbb {Z}), \end{aligned}$$

with the projection of $\pi _q (N)$ to the *i*th factor on the right being precisely $\pi _{p_i ^{a_i}} (N)$.

For (i), note that $l \le \log _2 q$, so it suffices to show that $Z_i = Z \big ( {{\,\textrm{SL}\,}}_V(\mathbb {Z}/p_i ^{a_i} \mathbb {Z}) \big )$ has order at most $|V |$. This last claim can be deduced from Hensel’s lemma: Since $Z_i$ consists of scalar matrices, its elements correspond to solutions to the equation $x^{|V |}=1$ in $\mathbb {Z}/p_i ^{a_i} \mathbb {Z}$, and these are distinct modulo $p_i$, as $|V|$ and $p_i$ are coprime.

For (ii), by Lemma 4.9 there exists *i* such that $\pi _{p_i ^{a_i}} (N)$ is non-central in ${{\,\textrm{SL}\,}}_V(\mathbb {Z}/p_i ^{a_i} \mathbb {Z})$. By projecting to this factor, we may therefore suppose that $q = p_i ^{a_i}$ and our claim is that $\pi _q (K_V (q/p_i)) \le \pi _q (N)$. This follows from the description of normal subgroups of ${{\,\textrm{SL}\,}}_V(\mathbb {Z}/p^a \mathbb {Z})$ given, for example, in [12] (see Theorem 3 and the Remarks following), noting that every nonzero ideal of $\mathbb {Z}/p^a \mathbb {Z}$ contains $p^{a-1}\mathbb {Z}/p^a \mathbb {Z}$. $\square$

Finally, we record an easy number-theoretic observation.

### Lemma 4.12

Let $m \ge 8$ and $q \in \mathbb {N}$. Then there exists a prime $p \in [m/4,m]$ and $r \mid q$ such that $(p,r)=1$ and $r \ge \sqrt{q}$.

### Proof

Recall Bertrand’s Postulate: for any $n \ge 4$, there exists a prime number in (*n*/2, *n*). So let $\tilde{p}$ be a prime in (*m*/2, *m*). Let $a \in \mathbb {N}$ be maximal such that $\tilde{p}^a \mid q$. If $\tilde{p}^a \le \sqrt{q}$, then take $\tilde{p} = p$ and $r = q/\tilde{p}^a$. If $\tilde{p}^a > \sqrt{q}$, then let *p* be any prime in (*m*/4, *m*) with $p \ne \tilde{p}$ (such *p* exist; for instance, any prime in (*m*/4, *m*/2) will do). Then let $b \in \mathbb {N}$ be maximal such that $p^b \mid q$, and let $r = q / p^b$. $\square$

We now prove Theorem 4.5. The key to the upper bound in (10) is the following construction. Henceforth, we write $B(n) = B_{S,\omega _0} (n)$.

### Proposition 4.13

Let $A(n) = \lbrace g \in E_{B(n)} (\mathbb {Z}): \Vert g \Vert _{\infty } \le 2^n \rbrace$; let $I(n) = A(n) \cdot B_S (n) \subseteq \mathcal {E}(\Gamma ,\Omega ,\mathbb {Z})$, and let $(\rho _n, \theta _n)$ be a local embedding of $\big ( B_S(n), B(n) \big )$ into $(Q_n,X_n)$. Then for any $q_n > 2^{n+1}$, there is a local embedding of *I*(*n*) into $\mathcal {E}(Q_n,X_n,\mathbb {Z}/q_n \mathbb {Z})$.

### Proof

Let $\tilde{\theta }_n:E_{B(n)}(\mathbb {Z})\rightarrow E_{X_n}(\mathbb {Z})$ be the injection induced by $\theta _n:B(n)\rightarrow X_n$. Note that for $A \in E_{B(n)}(\mathbb {Z})$, $\Vert \tilde{\theta }_n(A) \Vert _{\infty } = \Vert A \Vert _{\infty }$.

Let $\Phi _n: I(n) \rightarrow \mathcal {E}(Q_n,X_n,\mathbb {Z})$ be given by $\Phi _n(A \cdot g) = \tilde{\theta }_n(A)\cdot \rho _n(g)$. We claim that $\Phi _n$ is a local embedding. Given this claim, the conclusion easily follows: The congruence homomorphism $\pi _{q_n}: E_{X_n} (\mathbb {Z}) \rightarrow E_{X_n} (\mathbb {Z}/q_n \mathbb {Z})$ extends to a homomorphism $\pi _{q_n}:\mathcal {E}(Q_n,X_n,\mathbb {Z}) \rightarrow \mathcal {E}(Q_n,X_n,\mathbb {Z}/q_n \mathbb {Z})$ (which is the identity map on $Q_n$). Then $\pi _{q_n} \circ \Phi _n: I(n) \rightarrow \mathcal {E}(Q_n,X_n,\mathbb {Z}/q_n \mathbb {Z})$ is the desired local embedding: If $A_1,A_2 \in A(n)$ and $g_1,g_2 \in B_S(n)$, with:

$$\begin{aligned} \pi _{q_n}(\tilde{\theta }_n(A_1))\cdot \rho _n (g_1)&=(\pi _{q_n} \circ \Phi _n)(A_1 \cdot g_1) \\&=(\pi _{q_n} \circ \Phi _n)(A_2 \cdot g_2) \\&= \pi _{q_n}(\tilde{\theta }_n(A_2))\cdot \rho _n (g_2) \end{aligned}$$

then $g_1 = g_2$ and $\pi _{q_n}(\tilde{\theta }_n(A_1)) = \pi _{q_n}(\tilde{\theta }_n(A_2))$. The latter implies $\tilde{\theta }_n(A_1)= \tilde{\theta }_n(A_2)$ so that $A_1 = A_2$, since $\Vert \tilde{\theta }_n(A_i) \Vert _{\infty } \le 2^n$ and $q_n> 2^{n+1}$.

Therefore, let $A_1,A_2 \in A(n)$ and $g_1,g_2 \in B_S(n)$. Then $(A_1\cdot g_1)(A_2\cdot g_2) = \big ( A_1 A_2 ^{g_1 ^{-1}} \big )\cdot (g_1 g_2) \in I(n)$ iff (i) $g_1 g_2 \in B_S(n)$; (ii) $A_1 A_2 ^{g_1 ^{-1}} \in E_{B(n)}(\mathbb {Z})$; and (iii) $\Vert A_1 A_2 ^{g_1 ^{-1}} \Vert _{\infty } \le 2^n$. Since $A_1 \in E_{B(n)}(\mathbb {Z})$, (ii) is equivalent to (ii’) $A_2 \in \big ( E_{B(n)}(\mathbb {Z}) \big )^{g_1} = E_{B(n)g_1}(\mathbb {Z})$. Under these conditions:

$$\begin{aligned} \Phi _n \big ( (A_1 g_1)(A_2 g_2) \big )&= \tilde{\theta }_n (A_1)\tilde{\theta }_n (A_2 ^{g_1 ^{-1}}) \rho _n(g_1)\rho _n(g_2)\\ \Phi _n (A_1 g_1)\Phi _n (A_2 g_2)&=\tilde{\theta }_n (A_1)\tilde{\theta }_n (A_2)^{\rho _n(g_1) ^{-1}} \rho _n(g_1)\rho _n(g_2) \end{aligned}$$

so, writing $\tau _1 = \tilde{\theta }_n (A_2)^{\rho _n(g_1) ^{-1}}$ and $\tau _2 = \tilde{\theta }_n (A_2 ^{g_1 ^{-1}})$, it suffices to show that $\tau _1 = \tau _2$. We now conclude exactly as in the proof of Proposition 3.11, comparing the effect of $\tau _1$ and $\tau _2$ on the standard basis for the module $\mathbb {Z}^{X_n}$. $\square$

There is also a variant of the construction of Proposition 4.13 for $\mathcal {E}(\Gamma ,\Omega ,\mathbb {F}_p)$.

### Proposition 4.14

Let $A(n) = E_{B(n)} (\mathbb {F}_p)$, let $I(n) = A(n) \cdot B_S (n) \subseteq \mathcal {E}(\Gamma ,\Omega ,\mathbb {F}_p)$, and let $(\rho _n, \theta _n)$ be a local embedding of $\big ( B_S(n), B(n) \big )$ into $(Q_n,X_n)$. Then there is a local embedding of *I*(*n*) into $\mathcal {E}(Q_n,X_n,\mathbb {F}_p)$.

### Proof

This is much the same as the proof of Proposition 4.13, but easier: let $\tilde{\theta }_n:E_{B(n)}(\mathbb {F}_p)\rightarrow E_{X_n}(\mathbb {F}_p)$ be the injection induced by $\theta _n:B(n)\rightarrow X_n$, and let $\Phi _n: I(n) \rightarrow \mathcal {E}(Q_n,X_n,\mathbb {F}_p)$ be given by $\Phi _n(A \cdot g) = \tilde{\theta }_n(A)\cdot \rho _n(g)$. Then $\Phi _n$ is the desired local embedding, which is proved exactly as in Proposition 4.13. $\square$

### Proof of Theorem 4.5

First we prove (10). Let *A*(*n*), *I*(*n*), and $q_n$ be as in Proposition 4.13. We show that $B_{S \cup T(\omega _0)} (n) \subseteq I(n)$. The upper bound in (10) follows, as, taking $q_n \le \exp (n)$ (which we may) we have:

$$\begin{aligned} |\mathcal {E}(Q_n,X_n,\mathbb {Z}/q_n \mathbb {Z}) |&\le |{{\,\textrm{SL}\,}}_{X_n} (\mathbb {Z}/q_n \mathbb {Z}) |\cdot |Q_n |\\&\le q_n ^{|X_n |^2} \cdot |Q_n |\\&\le \exp \big ( n |X_n |^2 \big ) |Q_n |\text {.} \end{aligned}$$

Letting $C(S,T(\omega _0),n)$ be as in Lemma 3.10, it is enough to check that

$B_{C(S,T(\omega _0),n)}(n)\subseteq A(n)$. For $h \in C(S,T(\omega _0),n)$, there exist $s \in S$ and $g \in B_S (n-1)$ such that $h=E_{\omega _0,\omega _0 s} ^g=E_{\omega _0 g,\omega _0 sg}$ or $h=E_{\omega _0 s,\omega _0 } ^g=E_{\omega _0 sg,\omega _0 g}$. In either case, $h \in E_{B (n)} (\mathbb {Z})$, so $B_{C(S,T(\omega _0),n)} (m) \subseteq E_{B(n)}(\mathbb {Z})$ for all $m \in \mathbb {N}$. We claim, by induction, that for all $m \in \mathbb {N}$ and $g \in B_{C(S,T(\omega _0),n)}(m)$, $\Vert g \Vert _{\infty } \le 2^{m-1}$. This is clear for $m=1$. Letting $g \in B_{C(S,T(\omega _0),n)}(m)$ and supposing the claim holds for smaller *m*, write $g = g^{\prime } h$ for $g^{\prime } \in B_{C(S,T(\omega _0),n)}(m-1)$ and $h \in C(S,T(\omega _0),n)$, and let $v \in \Omega$. If $v \notin B(n)$, then $vg=v$, whereas if $v \in B(n)$ then by induction:

$$\begin{aligned} v g^{\prime } = \sum _{u \in B(n)} \lambda _u u \end{aligned}$$

for some $\lambda _u \in \mathbb {Z}$ with $|\lambda _u |\le 2^{m-2}$, so that there exist $w_1, w_2 \in B(n)$ such that $h^{\pm 1} = E_{w_1,w_2}$ and

$$\begin{aligned} vg = (v g^{\prime })h =(\pm \lambda _{w_1} +\lambda _{w_2}) w_2 +\sum _{u \ne w_2} \lambda _u u\text {.} \end{aligned}$$

Hence, $\Vert vg \Vert _{\infty } \le 2^{m-1}$, and since this holds for all *v*, $\Vert G \Vert _{\infty }\le 2^{m-1}$, as required.

For the lower bound, we apply Proposition 2.15 with $\mathcal {E}(\Gamma ,\Omega ,\mathbb {Z})$ replacing $\Gamma$ and $S \cup T(\omega _0)$ replacing *S*. Let $V_n = B_{S,\omega _0}(n)$, let $H_n = E_{V_n}(\mathbb {Z}) = {{\,\textrm{SL}\,}}_{V_n} (\mathbb {Z})$, let $G_n = (V_n,E_n)$ be as in Lemma 3.8, let $S^{\prime } _n = \lbrace E_{v,w}:v,w \in V_n, v\ne w \rbrace$ and let $S^{\prime \prime } _n = B_{S\cup T(\omega _0)} (4Cn^2)\cap H_n$, where $C>0$ is as in Proposition 4.3. This is permissable, since for $v,w \in V_n$ there exist $g,h\in B_S (n)$ with $v = \omega _0 g$ and $w = \omega _0 h$, so, using (8), and by the relation:

$$\begin{aligned} E_{v,w} = \big [ E_{v,\omega _0},E_{\omega _0,w} \big ]\, (\text {for}~ \omega _0 , v{\text { and }}w{\text { all distinct}}), \end{aligned}$$

$S^{\prime } _n \subseteq S^{\prime \prime } _n$, as per the conditions of Proposition 2.15. Since $S^{\prime \prime } _n \subseteq B_S (4Cn^2)$, we may clearly take $f(n) = 4Cn^2$. Taking $V=V_n$ in Theorem 2.21, we have $S^{\prime } _n = S_G$, so $H_n$ admits a finite presentation $\langle S^{\prime } _n \mid R_n \rangle$, with the relations $R_n$ being words of bounded length. We may therefore take *g*(*n*) to be a constant in Proposition 2.15, and conclude that for constants $C_2 \ge C_1 > 0$:

14 $$\begin{aligned} \mathcal {L}_{\mathcal {E}(\Gamma ,\Omega ,\mathbb {Z})} ^{S \cup T(\omega _0)} (C_2 n^2) \ge \mathcal {R}_{{{\,\textrm{SL}\,}}_{V_n}(\mathbb {Z})} ^{S_n ^{\prime \prime }}(C_1) \end{aligned}$$

(where $C_1 > 0$ may be chosen sufficiently large for our purposes). It therefore suffices to give a lower bound on the right-hand side of the last inequality. To this end, let *Q* be a finite group, and let $\phi : {{\,\textrm{SL}\,}}_{V_n} (\mathbb {Z}) \rightarrow Q$ be an epimorphism whose restriction to $B_{S_n ^{\prime \prime }} (C_1)$ is injective. For $n \ge 2$, $|V_n |\ge 3$, so by Theorem 4.8 there exists $q \in \mathbb {N}$ and a homomorphism $\psi : {{\,\textrm{SL}\,}}_{V_n}(\mathbb {Z}/q\mathbb {Z}) \rightarrow Q$ such that $\phi = \psi \circ \pi _q$, where $\pi _q: {{\,\textrm{SL}\,}}_{V_n}(\mathbb {Z}) \rightarrow {{\,\textrm{SL}\,}}_{V_n}(\mathbb {Z}/q\mathbb {Z})$ is the congruence map. We assume *q* to be the minimal natural number for which such a homomorphism $\psi$ exists.

First note that, for *m* larger than an absolute constant, $B_S (m)$ contains $S^{\prime } _2$, a generating set for ${{\,\textrm{SL}\,}}_{V_2}(\mathbb {Z})$. Thus, $B_{S^{\prime } _2} (4Cn^2/m) \subseteq S_n ^{\prime \prime }$, so $B_{S^{\prime } _2} (4C C_1 n^2/m) \subseteq B_{S_n ^{\prime \prime }} (C_1)$. Now ${{\,\textrm{SL}\,}}_{V_2}(\mathbb {Z})$ is a group of exponential growth, so there exists an absolute constant $C_3 > 0$ such that $|B_{S_n ^{\prime \prime }} (C_1 ) \cap {{\,\textrm{SL}\,}}_{V_2}(\mathbb {Z}) |\ge \exp (C_3 n^2)$. Now $\pi _q({{\,\textrm{SL}\,}}_{V_2}(\mathbb {Z}))={{\,\textrm{SL}\,}}_{V_2}(\mathbb {Z}/q\mathbb {Z})$, so by the injectivity of $\pi _q \mid _{B_{S_n ^{\prime \prime }} (C_1)}$ (and $|V_2 |\le 100 |S |^2$),

$$\begin{aligned} \exp (C_3 n^2) \le |{{\,\textrm{SL}\,}}_{V_2}(\mathbb {Z}/q\mathbb {Z}) |\le q^{100 |S |^2}. \end{aligned}$$

Hence, there is a constant $C_4 > 0$ such that $q \ge \exp (C_4 n^2)$. For *n* larger than an absolute constant, $|V_n |\ge 8$, so by Lemma 4.12 there exists a prime $|V_n |/ 4 \le p \le |V_n |$ such that there exist $a, r \in \mathbb {N}$ with $q = p^a r$, $(p,r)=1$ and $r \ge \sqrt{q} \ge \exp ((C_4 / 2) n^2)$. Let $W_n \subseteq V_n$ with $|W_n |= p$. Now let $N = \ker (\psi ) \vartriangleleft {{\,\textrm{SL}\,}}_{V_n}(\mathbb {Z}/q\mathbb {Z})$. By minimality of *q*, there is no proper divisor $q^{\prime }$ of *q* such that $\pi _q (K_{V_n}(q^{\prime })) \le N$. By Lemma 4.9, we may naturally identify ${{\,\textrm{SL}\,}}_{V_n}(\mathbb {Z}/q\mathbb {Z})$ with ${{\,\textrm{SL}\,}}_{V_n}(\mathbb {Z}/p^a\mathbb {Z}) \times {{\,\textrm{SL}\,}}_{V_n}(\mathbb {Z}/r\mathbb {Z})$. We claim that the restriction of $\psi$ to ${{\,\textrm{SL}\,}}_{W_n}(\mathbb {Z}/r\mathbb {Z})$ is injective. This yields the desired conclusion, since:

15 $$\begin{aligned} |Q |\ge |{{\,\textrm{SL}\,}}_{W_n}(\mathbb {Z}/r\mathbb {Z}) |\ge r^{C_5 |W_n |^2} \ge q^{C_5 |V_n |^2 /32} \ge \exp \big ( C_6 n^2 |V_n |\big ) \end{aligned}$$

for *n* sufficiently large and $C_5, C_6 > 0$ absolute constants. Taking *Q* of minimal order and applying (14), we have $\mathcal {L}_{\mathcal {E}(\Gamma ,\Omega ,\mathbb {Z})} ^{S \cup T} (C_2 n^3) \ge C_5 \exp \big ( C_6 n^3 |V_n |\big )$.

Suppose, then, that $N \cap {{\,\textrm{SL}\,}}_{W_n} (\mathbb {Z}/r\mathbb {Z})\ne 1$. By Proposition 4.11, there exists a prime divisor $p^{\prime }$ of *r* such that

$$\begin{aligned} K_{W_n} (r/p^{\prime }) \le N\cap {{\,\textrm{SL}\,}}_{W_n} (\mathbb {Z}/r\mathbb {Z}). \end{aligned}$$

Pulling back under the isomorphism from Lemma 4.9, we have $K_{W_n} (q/p^{\prime })\le N\le {{\,\textrm{SL}\,}}_{V_n}(\mathbb {Z}/q\mathbb {Z})$. By Proposition 4.10, $K_{V_n} (q/p^{\prime }) \le N$, so $\phi$ factors through $\pi _{q/p^{\prime }}$, contradicting minimality of *q*.

The proof of (11) is much the same, but easier. For the upper bound, let *I*(*n*) be as in Proposition 4.14. We show that $B_{S\cup T(\omega _0)}(n) \subseteq I(n)$ just as in the integer case (except without any concerns about the norms of matrices). By Proposition 4.14, we then have a local embedding of $B_{S\cup T(\omega _0)}(n)$ into $\mathcal {E}(Q_n,X_n,\mathbb {F}_p)$, which has order at most $p^{|X_n |^2} |Q_n |$. For the lower bound, we may once again take $S^{\prime } _n = \lbrace E_{v,w}:v,w \in V_n, v\ne w \rbrace$ and $S^{\prime \prime } _n = B_{S\cup T(\omega _0)} (4Cn^2)\cap H_n$, with $H_n = {{\,\textrm{SL}\,}}_{V_n} (\mathbb {F}_p)$, and deduce:

$$\begin{aligned} \mathcal {L}_{\mathcal {E}(\Gamma ,\Omega ,\mathbb {Z})} ^{S \cup T(\omega _0)} (C_2 n^2) \ge \mathcal {R}_{{{\,\textrm{SL}\,}}_{V_n}(\mathbb {F}_p)} ^{S_n ^{\prime \prime }}(C_1) \end{aligned}$$

for $C_2> C_1 > 0$, where we are able to choose $C_1$ sufficiently large. Since ${{\,\textrm{SL}\,}}_{V_n}(\mathbb {F}_p)$ is quasisimple whenever $|V_n |\ge 4$, we have:

$$\begin{aligned} \mathcal {R}_{{{\,\textrm{SL}\,}}_{V_n}(\mathbb {F}_p)} ^{S_n ^{\prime \prime }}(C_1) = |{{\,\textrm{PSL}\,}}_{V_n}(\mathbb {F}_p) |\ge c p^{|V_n |^2} \end{aligned}$$

for some $c>0$ depending only on *p*. $\square$

### Remark 4.15

LEF for $\mathcal {E}(\Gamma ,\Omega ,R)$ also holds for rings other than $\mathbb {Z}$ or $\mathbb {F}_p$. Indeed, for $\Gamma ,\Omega$ satisfying the conditions of Theorem 4.5, $\mathcal {E}(\Gamma ,\Omega ,R)$ is a LEF group whenever *R* is a *LEF ring*. We recall that a ring *R* is LEF if, for every finite subset $F \subseteq R$ there is a finite ring *A* and an injective map $\phi : F \rightarrow A$ such that for all $r,s \in F$, $r+s \in F$ implies $\phi (r+s)=\phi (r)+\phi (s)$ and $rs \in F$ implies $\phi (rs) = \phi (r)\phi (s)$. Apart from $\mathbb {Z}$, LEF rings include:

(i) Any finite ring;
(ii) The group ring $R[\Delta ]$, where *R* is a LEF ring and $\Delta$ is a LEF group.

Rings of type (ii) include the polynomial ring $R[t_1 ^{\pm 1}, \ldots t_k ^{\pm 1}]$ (respectively, $R\langle t_1 ^{\pm 1}, \ldots t_k ^{\pm 1}\rangle$) over *R* in *k* commuting (respectively, non-commuting) variables $t_1, \ldots , t_k$ and their multiplicative inverses, obtained by taking $\Delta = \mathbb {Z}^k$ (respectively, $\Delta = F_k$).

Moreover, in many cases we can prove an inequality redolent of the upper bound in (10). First we require the group $\mathcal {E}(\Gamma ,\Omega ,R)$ to be finitely generated. This will be the case, for instance, if there are finite subsets $T \subseteq R$, $U \subseteq R^{*}$, with $1 \in U$, such that *R* is generated as a $\mathbb {Z}$-module by:

$$\begin{aligned} \lbrace u_1 \cdots u_k t u_1 ^{\prime } \cdots u_k ^{\prime } : k \in \mathbb {N}; t \in T; u_i,u_i ^{\prime } \in U \rbrace \end{aligned}$$

(so that in particular, *R* is generated as a ring by $T \cup U \cup U^{-1}$). This condition is satisfied in examples (i) and (ii) (provided, in case (ii), the coefficient ring satisfies it too). To see that our condition implies finite generation of $\mathcal {E}(\Gamma ,\Omega ,R)$, note that for $r \in R$; $u \in R^{*}$ and $v,w,x \in \Omega$ distinct:

$$\begin{aligned} h_{v,x}(u) E_{v,w}(r) h_{v,x}(u)^{-1} = E_{v,w}(ur){\text { and }} h_{w,x}(u)^{-1} E_{v,w}(r) h_{w,x}(u) = E_{v,w}(ru), \end{aligned}$$

where

$$\begin{aligned} h_{y,z}(u) = E_{y,z} (u)E_{z,y} (-u^{-1})E_{y,z} (u)E_{y,z} (1)E_{z,y} (-1)E_{y,z} (1). \end{aligned}$$

Now we require a notion of LEF growth for rings. For $S \subseteq R$, a finite set generating *R* as a ring, with $0,1 \in S$, define $B_S (n)$ inductively by $B_S(1) = S$ and $B_S (n+1) = \lbrace sx+y: s\in S;x,y \in B_S(n) \rbrace$. The LEF growth $\mathcal {L}_R ^S (n)$ of *R* with respect to *S* is then the minimal size of a finite ring admitting a local embedding of $B_S (n)$. We note that, for $v, v_i, w_i \in \Omega$, and $s_i \in S$,

$$\begin{aligned} v E_{v_1,w_1} (s_1) \cdots E_{v_n,w_n} (s_n) = \lambda _0 v + \sum _i \lambda _i w_i \end{aligned}$$

for some $\lambda _i \in B_S(n)$. Arguing as in Theorem 4.5, we can conclude:

16 $$\begin{aligned} \mathcal {L}_{\mathcal {E}(\Gamma ,\Omega ,R)} (n) \preceq \mathcal {L}_R (n)^{|X_n |^2} |Q_n |\text {.} \end{aligned}$$

Finally, we can often usefully bound $\mathcal {L}_R (n)$ for finitely generated LEF rings *R*. For instance, $\mathcal {L}_{(\mathbb {Z},+,\cdot )} (n)\approx \exp (n)$; if *R* is finite then $\mathcal {L}_R (n)$ is bounded, while, if *R* is a LEF ring generated by the finite set *X* and $\Delta$ is a LEF group generated by the finite set *S*, then $R[\Delta ]$ is generated by $X \cup S$, and $B_{X \cup S} (n)$ consists of combinations with coefficients in $B_X(n)$ and supported on $B_S(n)$. As such, if $B_X(n)$ and $B_S(n)$ admit local embeddings into *A* and *Q*, respectively, then $B_{X \cup S} (n)$ admits a local embedding into *A*[*Q*], and consequently, $\mathcal {L}_{R[\Delta ]} ^{X \cup S} (n) \le \mathcal {L}_R ^X (n) ^{\mathcal {L}_{\Delta } ^S (n)}$.

## The diversity of LEF growth functions

### Definition 5.1

A strictly increasing function $f: \mathbb {N} \rightarrow \mathbb {N}$ is *permissible* if (a) $f(0) = 1$; (b) There exists $D \in \mathbb {N}$ such that for all $n \ge 1$,

17 $$\begin{aligned} f(n+1)-f(n) \le D \big (f(n)-f(n-1)\big )\text {.} \end{aligned}$$

Fix a free basis $S_{2d} = \lbrace a_1, b_1, \ldots , a_d, b_d \rbrace$ for the free group $F_{2d}$ of rank 2*d*.

### Proposition 5.2

For any permissible function *f*, there exist $d \in \mathbb {N}$; a set $\Omega (f)$; a transitive action $\Omega (f) \curvearrowleft F_{2d}$; a point $\omega _0 \in \Omega (f)$; finite groups $Q_n$ and transitive actions $X_n \curvearrowleft Q_n$, such that for all $n \in \mathbb {N}$,

(i) $$|B_{S_{2d},\omega _0} (n) |= f(n)$$;
(ii) There exists a local embedding of $\big ( B_{S_{2d}}(n),B_{S_{2d},\omega _0} (n) \big )$ into $(Q_n,X_n)$;
(iii) $$|X_n |= f(2n)$$;
(iv) $|Q_n |\le f(2n)! \exp (Cn)$, for some absolute constant $C>0$.

### Remark 5.3

Proposition 5.2 (i) shows that every permissable function arises as the growth function of a Schreier graph of a transitive action of a finitely generated group, and in particular of a connected graph of bounded vertex valence. Conversely, if $G=(V,E)$ is an infinite connected graph with every vertex having valence at most *D*, and for any $v_0 \in V$, the number *f*(*n*) of vertices of *G* at distance at most *n* from $v_0$ is a permissable function.

### Proof of Theorem 1.1

Let $\Omega (f) \curvearrowleft F_{2d}$; $\omega _0 \in \Omega (f)$ and $X_n \curvearrowleft Q_n$ be as in Proposition 5.2. Let $\Delta (f) = \mathcal {S} (F_{2d}, \Omega (f))$. Then by the inequalities (2) from Theorem 3.3, and by Proposition 5.2 (ii),

$$\begin{aligned} \gamma _{F_{2d},\Omega (f)} (n)! \preceq \mathcal {L}_{\Delta (f)} (n) \preceq |X_n |! |Q_n |. \end{aligned}$$

On the one hand, by Proposition 5.2 (i),

$$\begin{aligned} f(n)! \preceq \gamma _{F_{2d},\Omega (f)} (n)!. \end{aligned}$$

On the other hand, by Proposition 5.2 (iii) and (iv),

$$\begin{aligned} |X_n |! |Q_n |&\le f(2n)! f(2n) \exp (Cn) \\&\preceq \exp \big ( f(2n) \log f(2n)+ \log f(2n) + Cn \big ) \\&\preceq \exp \big ( f(n)\log f(n) \big ) {\text { (since }}n \preceq f(n)\text {)}\\&\preceq f(n)! \end{aligned}$$

(using Stirling’s approximation). $\square$

It remains to prove Proposition 5.2. First we construct the set $\Omega (f)$ and the action of $F_{2d}$ upon it.

### Proposition 5.4

Let $f: \mathbb {N} \rightarrow \mathbb {N}$ be a permissable function. There exist $d \ge 1$; a set $\Omega (f)$; a transitive action $\Omega (f) \curvearrowleft F_{2d}$ and a point $\omega _0 \in \Omega (f)$ such that for all $n \in \mathbb {N}$, $B_{S_{2d},\omega _0} (n) = f(n)$ and moreover, letting $L_0 = \lbrace \omega _0 \rbrace$ and for $n \ge 1$ taking $L_n = B_{S_{2d},\omega _0} (n) {\setminus } B_{S_{2d},\omega _0} (n-1)$, we have for $1 \le i \le d$:

(i) $a_i$ preserves $L_{2m} \sqcup L_{2m+1}$ for each $m \ge 0$;
(ii) $b_i$ preserves $L_{2m-1} \sqcup L_{2m}$ for each $m \ge 1$ and fixes $\omega _0$.

### Proof

Let $D \in \mathbb {N}$ be such that *f* satisfies (17) for all $n \ge 1$. Let $d \in \mathbb {N}$ with $2(d-1) \ge D$. Let $\lbrace L_n: n \in \mathbb {N} \rbrace$ be a family of disjoint finite sets, with $L_0 = \lbrace \omega _0 \rbrace$ and $|L_n |= f(n)-f(n-1)$ for $n \ge 1$. Set $\Omega (f)$ to be the disjoint union of the $L_n$.

Let us define the action of the $a_i$ on $L_{2m} \sqcup L_{2m+1}$. First suppose that $|L_{2\,m+1} |\le |L_{2\,m} |$. Let $U \subseteq L_{2\,m}$ with $|U |= |L_{2\,m+1} |$. Define $a_1$ on $L_{2m} \sqcup L_{2m+1}$ to be the product of $|U |$ disjoint transpositions, each supported on one point of *U* and one point of $L_{2m+1}$ (and all points of $L_{2m} \setminus U$ are fixed). Let $a_2, \ldots , a_d$ act trivially on $L_{2m} \sqcup L_{2m+1}$.

Now suppose instead that $|L_{2\,m+1} |> |L_{2\,m} |$. Write $|L_{2\,m+1} |= 2j |L_{2\,m} |+ k$, for some $0 \le j \le d-1$ and $0 \le k \le 2 |L_{2\,m} |$. Partition $L_{2m+1}$ as:

$$\begin{aligned} L_{2m+1} = W \sqcup \bigsqcup _{i=1} ^j V_i \end{aligned}$$

with $|V_i |= 2 |L_{2\,m} |$ and $|W |= k$. For $1 \le i \le j$, define $a_i$ on $L_{2\,m} \sqcup L_{2\,m+1}$ to be the product of $|L_{2\,m} |$ disjoint 3-cycles, each supported on one element of $L_{2m}$ and two elements of $V_i$. Write $k = k_1 + 2 k_2$ for some $k_1, k_2 \in \mathbb {N}$ with $0 \le k_1 + k_2 \le |L_{2\,m} |$. Partition $L_{2\,m}$ and *W* as:

$$\begin{aligned} L_{2m} = U_0 \sqcup U_1 \sqcup U_2 \text { and } W = W_1 \sqcup W_2 \end{aligned}$$

where $|U_1 |= |W_1 |= k_1$ and $2 |U_2 |= |W_2 |= 2 k_2$. Define $a_{j+1}$ on $L_{2\,m} \sqcup L_{2\,m+1}$ to be the product of $k_1$ disjoint transpositions and $k_2$ disjoint 3-cycles, with each transposition supported on one point each of $U_1$ and $W_1$, and each 3-cycle supported on one point of $U_2$ and two points of $W_2$ (with all points of $U_0$ being fixed). If $j \le d-2$, then let $a_{j+2}, \ldots , a_d$ act trivially on $L_{2m} \sqcup L_{2m+1}$.

The $b_i$ are defined on $L_{2m-1} \sqcup L_{2m}$ in a precisely analogous way, with $L_{2\,m-1}$ and $L_{2\,m}$, respectively, replacing $L_{2\,m}$ and $L_{2\,m+1}$ in the above construction (and $\omega _0$ being fixed by all $b_i$). Certainly the $a_i$ and $b_i$ act as well-defined permutations of $\Omega (f)$ satisfying (i) and (ii). By construction, for $n \ge 1$ every element of $L_n$ is adjacent in the Schreier graph to at least one element each from $L_{n-1}$, but to no points outside $L_{n-1} \cup L_n \cup L_{n+1}$. Thus, the elements of $L_n$ are precisely the vertices of the Schreier graph at distance *n* from $\omega _0$. $\square$

### Proof of Proposition 5.2

Let *d*, $\Omega (f) \curvearrowleft F_{2d}$ and $\omega _0 \in \Omega (f)$ be as in Proposition 5.4, and write *S* for $S_{2d}$. Suppose $n \ge 2$. Let $X_n = B_{S,\omega _0} (2n)$; let $\theta _n: B_{S,\omega _0} (n) \rightarrow X_n$ be inclusion. For $1 \le i \le d$ we define $\alpha _{n,i}, \beta _{n,i} \in {{\,\textrm{Sym}\,}}(X_n)$ such that $(x)\alpha _{n,i} = (x)a_i$ for $x \in B_{S,\omega _0} (2n-1)$; the restriction of $\alpha _{n,i}$ to $L_{2n}$ is an arbitrary permutation of $L_{2n}$ and $(x)\beta _{n,i} = (x)b_i$ for $x \in X_n$. By the construction of the $L_m$ in Proposition 5.4, $\alpha _{n,i}$ and $\beta _{n,i}$ are well-defined permutations of $X_n$. Let $P_n = \langle \alpha _{n,i}, \beta _{n,i}: 1 \le i \le d \rangle \le {{\,\textrm{Sym}\,}}(X_n)$. There is a unique homomorphism $\phi _n: F_{2d} \rightarrow P_n$ satisfying $\phi (a_i) = \alpha _{n,i}$ and $\phi (b_i) = \beta _{n,i}$ for $1 \le i \le d$.

By Example 2.18 (iv), there exists a constant $C>0$ and finite groups $G_n$ such that for every *n*, $|G_n |\le \exp (Cn)$ and there is a homomorphism $\psi _n: F_{2d} \rightarrow G_n$ whose restriction to $B_S (n)$ is injective. Let $Q_n = P_n \times G_n$, and let $Q_n$ act on $X_n$ via $(x) (\sigma ,g) = (x)\sigma$. Then $Q_n$ and $X_n$ satisfy conclusions (iii) and (iv). Further, $\pi _n = (\phi _n \times \psi _n): F_{2d} \rightarrow Q_n$ is a homomorphism whose restriction to $B_S (n)$ is injective.

We claim that the restriction of $\pi _n$ to $B_S (n)$ and $\theta _n$ satisfy condition (1) of Definition 2.7. Given the above, this will imply that $(\pi _n,\theta _n)$ yields a local embedding of actions, and will conclude the proof. Let $g \in B_S (n)$ be a word of length at most *n*, and let $\omega \in B_{S,\omega _0} (n)$. Suppose that $\omega g \in B_{S,\omega _0} (n)$. Then there is a path in ${{\,\textrm{Schr}\,}}(F_2,\Omega (f),S)$ from $\omega$ to $\omega g$ of length at most *n*. This path lies entirely inside $B_{S,\omega _0} (3n/2) \subseteq B_{S,\omega _0} (2n-1)$ (since $n \ge 2$). By construction of the $\alpha _{n,i}$ and $\beta _{n,i}$, the induced subgraphs on $B_{S,\omega _0} (2n-1)$ and $B_{\pi _n(S),\omega _0} (2n-1)$ in ${{\,\textrm{Schr}\,}}(F_2,\Omega (f),S)$ and ${{\,\textrm{Schr}\,}}(P_n,X_n,\pi _n(S))$ are isomorphic, with $a_i$-labeled edges (respectively, $b_i$-labeled edges) corresponding to $\alpha _{n,i}$-labeled edges (respectively, $\beta _{n,i}$-labeled edges). Thus, $\theta _n (\omega ) \pi _n (g) = \omega g = \theta _n (\omega g)$, as required. $\square$

### Proof of Corollary 1.2

In cases (i), (ii) and (iii), we take $\tilde{f}(n) = n^{\alpha }/\log (n)$, $\exp (\log (n)^{\alpha }) / \log (n)^{\alpha }$ and $\exp (n^{\beta })/n^{\beta }$, so that $\tilde{f}(n) \log \tilde{f}(n) \approx n^{\alpha }$, $\exp (\log (n)^{\alpha })$ and $\exp (n^{\beta })$, respectively. Then $\tilde{f}$ satisfies condition (17) for all *n* sufficiently large, and consequently there exists a permissable function *f* such that $f \approx \tilde{f}$. Applying Theorem 1.1 to *f* and using Stirling’s approximation, there exist $C,c>0$ such that

$$\begin{aligned} \exp \big ( c \tilde{f}(n) \log \tilde{f}(n) \big ) \preceq \mathcal {L}_{\Delta (f)} \preceq \exp \big ( C \tilde{f}(n) \log \tilde{f}(n) \big ) \end{aligned}$$

as required. $$\square $$

## Data Availability

Data sharing is not applicable to this article as no datasets were generated or analyzed during the current study.
